# Inhibition of DEF‐p65 Interactions as a Potential Avenue to Suppress Tumor Growth in Pancreatic Cancer

**DOI:** 10.1002/advs.202401845

**Published:** 2024-05-17

**Authors:** Sicong Huang, Jiaqi Yang, Teng Xie, Yangwei Jiang, Yifan Hong, Xinyuan Liu, Xuyan He, Damiano Buratto, Dong Zhang, Ruhong Zhou, Tingbo Liang, Xueli Bai

**Affiliations:** ^1^ Department of Hepatobiliary and Pancreatic Surgery the First Affiliated Hospital Zhejiang University School of Medicine Hangzhou 310000 China; ^2^ Institute of Quantitative Biology, College of Life Sciences Zhejiang University Hangzhou 310000 China; ^3^ Key Laboratory of Pancreatic Disease of Zhejiang Province Hangzhou 310000 China; ^4^ Innovation Center for the Study of Pancreatic Diseases of Zhejiang Province Hangzhou 310000 China; ^5^ Zhejiang Clinical Research Center of Hepatobiliary and Pancreatic Diseases Hangzhou 310000 China; ^6^ Shanghai Institute for Advanced Study Zhejiang University Shanghai 200000 China; ^7^ Life Sciences Institute Zhejiang University Hangzhou 310000 China; ^8^ Department of Chemistry Columbia University New York 10027 USA; ^9^ Cancer Center Zhejiang University Hangzhou 310000 China

**Keywords:** computational design, digestive organ expansion factor, p65, pancreatic ductal adenocarcinoma, peptide therapeutics

## Abstract

The limited success of current targeted therapies for pancreatic cancer underscores an urgent demand for novel treatment modalities. The challenge in mitigating this malignancy can be attributed to the digestive organ expansion factor (DEF), a pivotal yet underexplored factor in pancreatic tumorigenesis. The study uses a blend of in vitro and in vivo approaches, complemented by the theoretical analyses, to propose DEF as a promising anti‐tumor target. Analysis of clinical samples reveals that high expression of DEF is correlated with diminished survival in pancreatic cancer patients. Crucially, the depletion of DEF significantly impedes tumor growth. The study further discovers that DEF binds to p65, shielding it from degradation mediated by the ubiquitin‐proteasome pathway in cancer cells. Based on these findings and computational approaches, the study formulates a DEF‐mimicking peptide, peptide‐031, designed to disrupt the DEF‐p65 interaction. The effectiveness of peptide‐031 in inhibiting tumor proliferation has been demonstrated both in vitro and in vivo. This study unveils the oncogenic role of DEF while highlighting its prognostic value and therapeutic potential in pancreatic cancer. In addition, peptide‐031 is a promising therapeutic agent with potent anti‐tumor effects.

## Graphical abstract

The study identifies the digestive organ expansion factor (DEF) as a key player in pancreatic cancer, correlating high DEF expression with poor patient survival. DEF interacts with p65, preventing it from ubiquitination‐induced degradation and promoting tumor cell survival. Peptide‐031, designed to disrupt this interaction, effectively inhibits tumor growth in vitro and in vivo, offering a novel therapeutic approach with significant anti‐tumor potential.

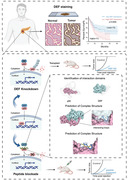

## 1 Introduction

Pancreatic cancer is a leading cause of cancer‐related mortality globally. The incidence of pancreatic cancer has risen drastically over the past decades.^[^ ^1^, ^2^, ^3^ ^]^ Surgical intervention is only feasible for 10–15% of patients with localized disease, most incidences initially necessitate neoadjuvant or palliative systemic treatment.^[^ ^4^ ^]^ Targeted therapy holds promise in achieving long‐term disease control with reduced systemic toxicity. Despite the approval of targeted therapies for pancreatic cancer,^[^ ^5^, ^6^ ^]^ their overall survival benefits remain limited. For instance, the Pancreas Olaparib Ongoing (POLO) trial demonstrated improved progression‐free survival through olaparib, a poly (ADP‐ribose) polymerase inhibitor (PARPi), in metastatic patients harboring germline *BRCA1/2* variations.^[^ ^7^, ^8^ ^]^ Although efforts have been made to develop treatments targeting low‐frequency variants such as Tyrosine Kinase Receptor (*TRK*) fusion (<1%),^[^ ^9^ ^]^ Neuregulin 1 (*NRG1)*  fusion (<1%),^[^ ^10^ ^]^ and Kirsten Rat Sarcoma Viral oncogene (*KRAS^G12C^*) mutation (≈2%)^[^ ^11^, ^12^ ^]^; progress in the development of targeted therapies has been hindered by small patient cohorts enrolled in these trials. Therefore, enhanced and broader therapeutic targets for pancreatic cancer need to be identified urgently.

The digestive organ expansion factor (DEF), also known as U3 small nucleolar RNA‐associated protein 25 homolog (UTP25), is an integral component of the ribosomal small subunit (SSU) processome. It is involved in the initial stages of 40S ribosomal subunit assembly and facilitates the modification of 18S ribosomal RNA.^[^ ^13^, ^14^ ^]^ DEF is indispensable for digestive organogenesis and was first identified in zebrafish. Mutations in DEF result in a small liver phenotype, along with a reduction in pancreas size and thinning of the intestine.^[^ ^15^ ^]^ The loss of DEF function leads to hypoplasia of digestive organs.^[^ ^16^ ^]^ Research has indicated that DEF facilitates the degradation of the p53 protein through a mechanism that is independent of the proteasome pathway.^[^ ^17^ ^]^ As a critical tumor suppressor, the mutations in p53 have been observed in a significant proportion of pancreatic cancer cases and are considered a hallmark of the disease.^[^ ^18^, ^19^ ^]^ Instead of acting as a guardian of the genome, mutant p53 can acquire oncogenic properties, thereby disrupting key cellular processes and contributing to the initiation and progression of pancreatic cancer.^[^ ^20^, ^21^ ^]^ However, the precise role of DEF in the progression of pancreatic cancer, along with its potential interactions with pathways associated with pancreatic oncogenesis in humans, continue to be subjects of significant interest. Additionally, the specific vulnerability of the DEF‐mediated pathways in pancreatic cancer, compared to healthy pancreatic tissues, requires further elucidation.

The NF‐κB signaling pathway has been implicated in the development and progression of pancreatic cancer, and p65, a subunit of the NF‐κB transcription factor complex, plays a crucial role in this process.^[^ ^22^, ^23^ ^]^ The activation of p65 has been associated with various detrimental effects in pancreatic cancer, including genetic alterations, metabolic changes, acquisition of cancer stem cell properties, epithelial‐to‐mesenchymal transition, invasion, angiogenesis, metastasis, therapy resistance, and suppression of anti‐tumor immunity.^[^ ^24^, ^25^ ^]^ Therefore, inhibition of p65 activation in cancer appears to be a promising option for improving the efficacy of conventional anti‐cancer therapies with the potential for minimal side effects.^[^ ^26^, ^27^ ^]^

**Figure 1:**
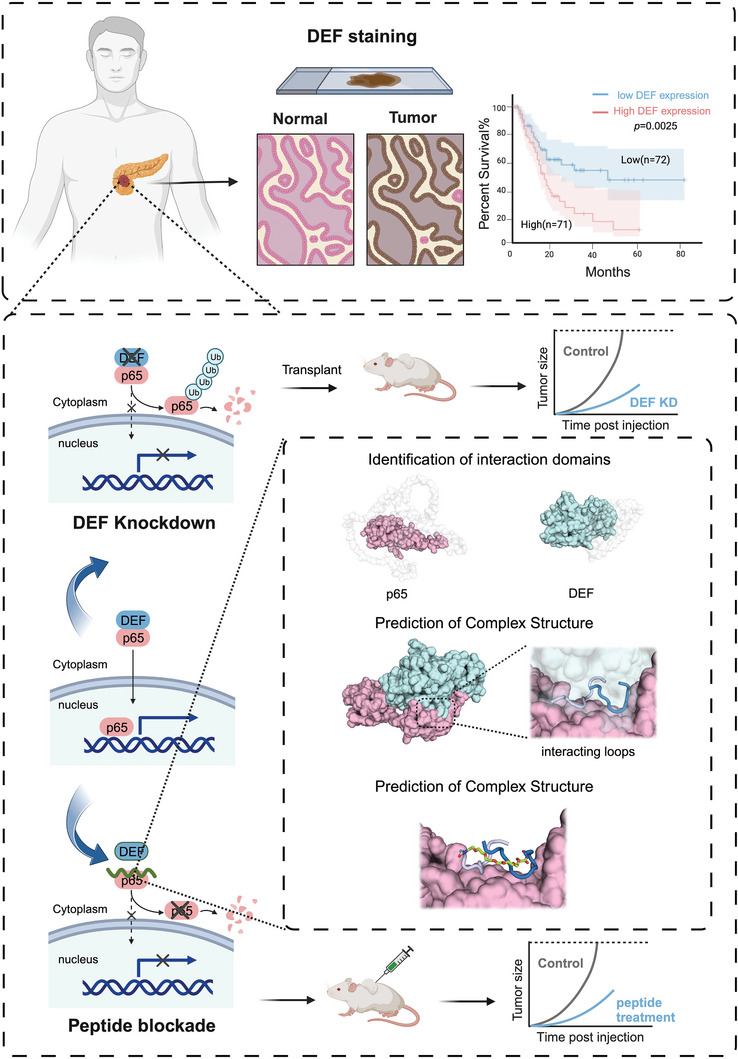
Schematic illustration of the design and mechanism of DEF‐mimicking peptide for anti‐tumor therapy in pancreatic cancer. DEF exhibits high expression levels in pancreatic cancer and is associated with poor prognosis. The depletion of DEF leads to profound inhibition of cell proliferation and inhibits tumor progression in pancreatic cancer. Notably, DEF interacts with p65 and enhances its stability by preventing ubiquitination‐mediated degradation in pancreatic cancer cells. Consequently, a DEF‐ mimicking peptide was developed with the specific aim of targeting p65 and exerting remarkable anti‐tumor effects in pancreatic cancer.

In this study, we comprehensively investigated the role of DEF in pancreatic cancer progression and its potential as a novel target for anti‐tumor therapy. Through a series of in vitro and in vivo experiments, along with theoretical calculations, we systematically explored the significance of DEF in tumor progression and its potential as a promising therapeutic target. Our analysis of clinical samples, followed by several experimental validations, confirmed that DEF plays a crucial role in promoting pancreatic cancers and shows potential as an effective therapeutic target. Importantly, we observed that the depletion of DEF inhibits the NF‐κB pathway without impacting the p53 pathway. Mechanistically, we identified specific binding sites where DEF forms a complex with the p65 protein. This complex stabilizes p65, preventing ubiquitination‐induced degradation and promoting tumor cell survival. Based on these findings, we mimicked this binding mode and designed a blocking peptide (peptide‐031) that targeted p65 and disrupted the critical interaction between DEF and p65. Subsequent in vitro and in vivo experiments demonstrated that treating pancreatic cancer cells with peptide‐031 effectively downregulated p65 expression and inhibited tumor growth. Overall, the study findings highlight the oncogenic role of DEF in pancreatic cancer while establishing it as a valuable therapeutic target for intervention (**Figure** **1**).

## 2 Results

### 2.1 Expression of DEF is Significantly Upregulated in Pancreatic Cancer and Correlated with Unfavorable Prognosis

**Figure 2:**
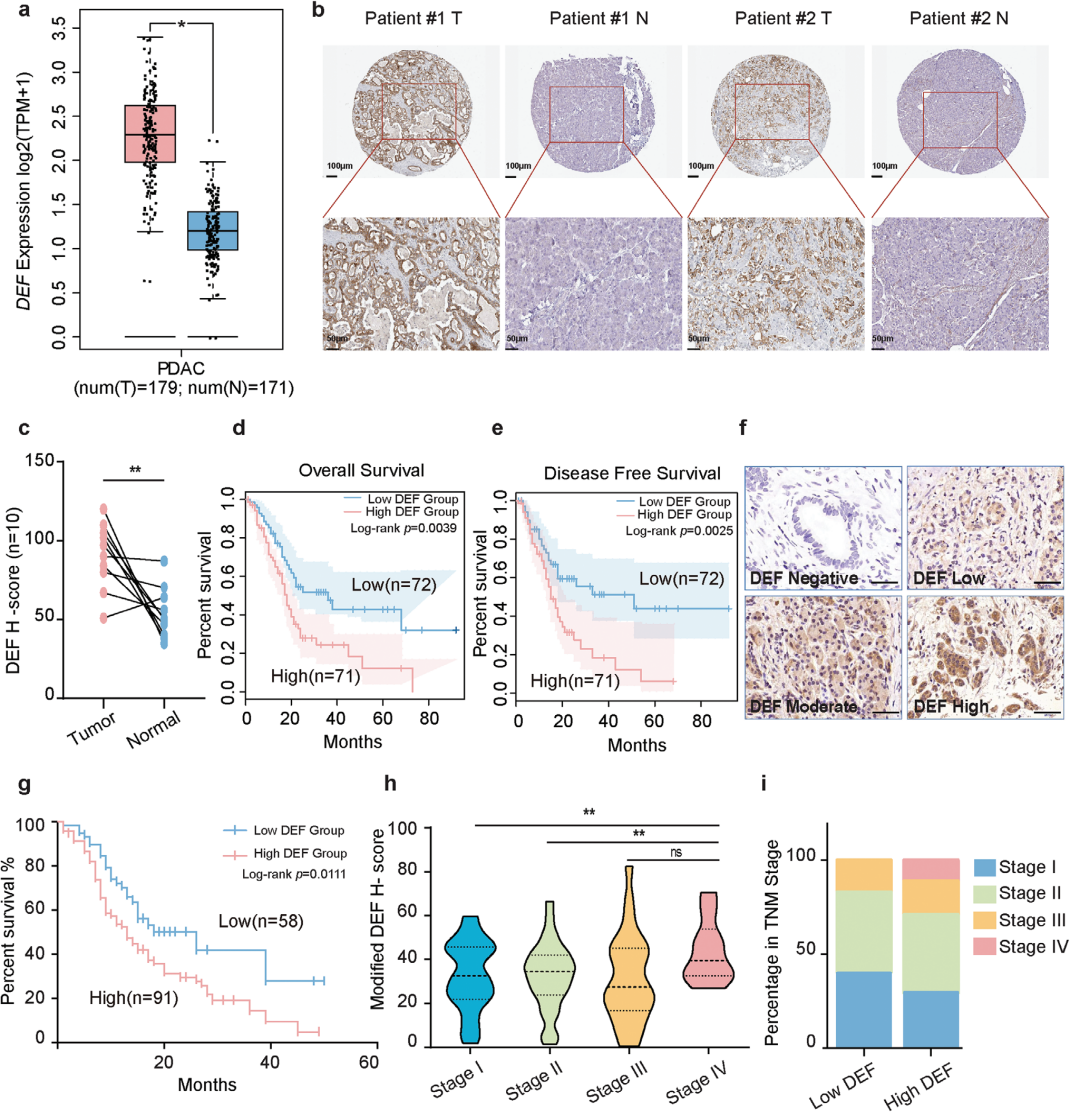
DEF is highly expressed in pancreatic ductal adenocarcinoma (PDAC) and is associated with poor prognosis. a) TCGA data analysis showed the differential expression profile of DEF (*n* = 179 patients for pancreatic cancers; *n* = 171 patients for normal pancreas) in paired tumor and normal pancreatic tissues. The relative DEF expression in pancreatic cancer was analyzed using large‐scale RNA‐Seq datasets of PDAC from the TCGA database. b,c) DEF expression was measured in paired tumor and normal pancreatic tissue by IHC staining with representative images (scale bars: 100 µm) (b) and statistical results were shown (*n* = 10) (c). d) Kaplan–Meier survival revealed the association of *DEF* mRNA abundance with the survival of pancreatic cancer patients. The log‐rank *p* values are shown. Overall survival (OS) of patients with pancreatic cancer with high or low concentrations of DEF (*n* = 143). e) Disease free survival (DFS) of patients with pancreatic cancer with high or low concentrations of DEF (*n* = 143). f) Representative images of DEF immunostaining in the pancreatic cancer tissue microarray, with 149 pancreatic cancer samples. Scale bars: 50 µm. DEF IHC staining grades were quantified by multiplying the intensity of the signal with the percentage of positive cells (negative, low, moderate, and high groups). The IHC staining of the tumor was categorized as low (including negative and low groups, *n* = 58) and high (including moderate and high groups, *n* = 91). g) Tissue microarray analysis of the prognostic role of DEF in pancreatic cancer. h) Modified quantitative H‐scores of DEF between pancreatic tumor tissues with different clinical stages in the pancreatic cancer tissue microarray, with 149 pancreatic cancer samples. i) The association with DEF levels in pancreatic cancers and TNM stages in multiple patient cohorts of pancreatic cancer tissue microarray. ns, not significant, ^*^ *p* < 0.05, ^**^ *p* < 0.01 according to a two‐tailed *t*‐test. All data are representative of three independently performed experiments.

To assess the clinical significance of DEF in pancreatic cancer, we conducted a bioinformatic analysis using data from The Cancer Genome Atlas (TCGA) public database. Compared to normal pancreatic tissues, tumor samples exhibited significantly elevated expression levels of DEF (**Figure** **2**a), which was consistent with the results of protein‐level analysis of clinical samples (*n* = 149) obtained from patients with pancreatic ductal adenocarcinoma (PDAC) and their paired normal tissues (Figure 2b,c; Figure S1a, Supporting Information). In addition, Kaplan‐Meier analysis showed that higher DEF expression was associated with poorer overall and disease‐free survival in patients with PDAC from the TCGA database (Figure 2d,e). Immunohistochemistry (IHC) analysis of a PDAC tissue microarray containing the 149 paired clinical samples confirmed the upregulation of DEF in tumor tissues (Figure 2f), further revealing that patients with higher DEF expression levels had reduced survival compared to those with lower levels (Figure 2g). Furthermore, an examination of clinical characteristics indicated a correlation between increased abundance of DEF and advanced pathological tumor stage. Notably, patients in advanced stages exhibited higher levels of DEF expression (Figure 2h,i). The results of the tissue microarray (Table S1, Supporting Information) indicate that DEF was also associated with the tumor‐node‐metastasis (TNM) stage, lymphatic and distant metastases. Collectively, these findings establish a significant correlation between DEF expression and prognosis in pancreatic cancer.

### 2.2 Depletion of DEF Significantly Impedes Cellular Proliferation and Neoplastic Progression in Pancreatic Cancer

**Figure 3:**
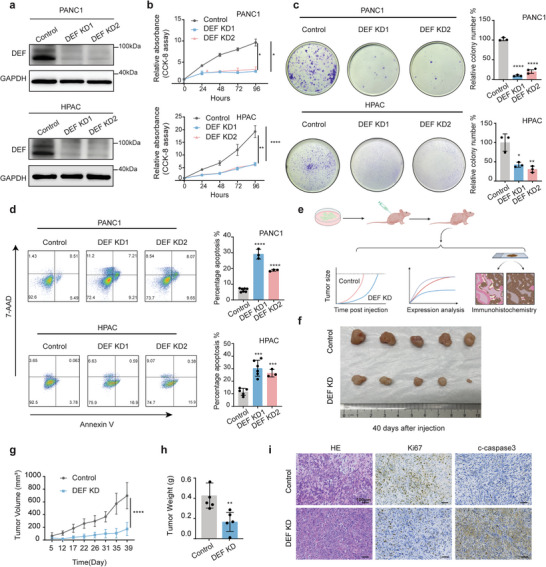
DEF is required for tumorigenesis. a) Western blot analysis of the indicated proteins in control and DEF knockdown cells. b) Cell Counting Kit‐8 assay comparing control and DEF knockdown cells are shown. c) Colony formation and statistical analysis of PANC1 and HPAC cells. d) Flow cytometric analysis was used to evaluate the incidence of apoptosis after DEF knockdown in PANC1 and HPAC cells. e) Schematic protocols displaying the xenograft tumors after subcutaneous injections into immunodeficient mice with control and DEF‐depleted pancreatic cancer cells. f–h) Representative images of tumors (*n* = 5) (f), g) tumor volumes, and h) tumor weights were measured in the indicated groups. Tumors were measured at specified time points and dissected at the endpoints (*n* = 5). i) Representative images of the results of the H&E staining, the IHC staining of Ki 67, and cleaved‐caspase 3 in DEF knockdown tumors within immunodeficient nude mice. Scale bars: 100 µm. Results are presented as mean ± SD. ^*^ *p* < 0.05, ^**^ *p* < 0.01, ^***^ *p* < 0.001 according to a two‐tailed *t*‐test. All data are representative of three independently performed experiments.

To evaluate the relevance of DEF in pancreatic cancer, we selected distinct cell lines: PANC1, a human PDAC cell line harboring a p53 mutation; HPAC, a human PDAC cell line expressing wild‐type p53; and KPC, a cell line derived from *KRAS ^LSL‐G12D^;TP53^LSL‐R172H^; Pdx1‐Cre* (KPC) mice with PDAC. After DEF knockdown (KD) (**Figure** **3**a; Figure S1b, Supporting Information), we observed that the depletion of DEF in both mutant and wild‐type p53 cells significantly suppressed the proliferation of PDAC cells (Figure 3b,c; Figure S1c,d, Supporting Information). Consistently, the downregulation of DEF expression led to substantial inhibition of cell growth as determined by an EdU assay in PDAC cells (Figure S1e, Supporting Information). The percentage of DEF KD cells undergoing apoptosis was higher than that of control cells as depicted in Figure 3d and Figure S1f (Supporting Information). Furthermore, pancreatic cancer cell lines with different p53 mutation statuses exhibited similar phenotypes following DEF KD. To further investigate the oncogenic role of DEF in pancreatic cancer progression, control and DEF KD pancreatic cancer cells were subcutaneously injected into nude mice (Figure 3e). The results revealed a significant reduction in xenograft tumor growth in mice injected with DEF KD cells compared to those injected with control cells for up to 40 days post‐injection. The reduction in tumor volume and weight was significantly greater in the DEF KD group (Figure 3f–h; Figure S1g–i, Supporting Information). Additionally, DEF KD tumors exhibited decreased levels of Ki67 expression, while displaying increased cleaved caspase 3 expression in contrast with control tumors (Figure 3i). Therefore, our findings substantiate the role of DEF in fostering tumor progression in pancreatic cancer.

### 2.3 DEF Interacts with p65 to Inhibit its Ubiquitination‐Mediated Degradation in Pancreatic Cancer Cells, While Exerting no Effect on p53

**Figure 4:**
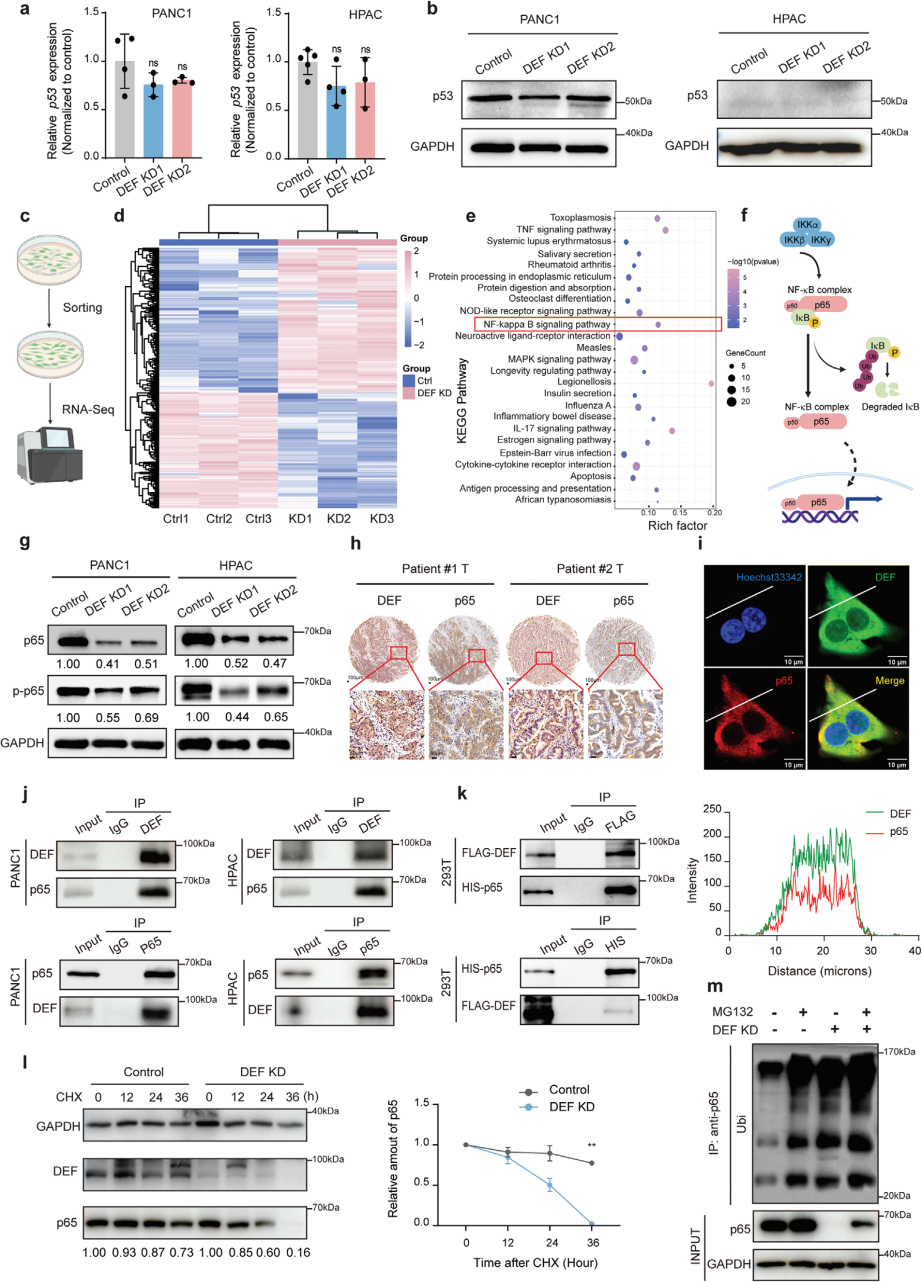
DEF positively correlates and interacts with p65 in pancreatic cancer. a) RT‐qPCR analysis of *p53* in control and *DEF* knockdown PANC1 and HPAC cells is shown. b) Western blot analysis of p53 expression is shown in control and DEF knockdown PANC1 and HPAC cells. c) Schematic diagram of RNA‐sequence in control (*n* = 3) relative to *DEF* knockdown (*n* = 3) PANC1 cells. d) Expression heatmaps depict the genes that were downregulated and upregulated in the comparisons between the control and DEF knockdown groups (Fold change >1.5, *p* < 0.05). e) Kyoto Encyclopedia of Genes and Genomes analysis shows the top 20 downregulated hallmark pathways. f) Pathway diagram of the classical NF‐κB signaling. g) Western blot analysis of p65 and p‐p65 with control and DEF knockdown in PANC1 and HPAC cells is shown. h) Representative images of IHC staining of DEF and p65 in a tissue microarray. Scale bars: 100 µm. i) Immunofluorescence analysis showing co‐localization of DEF with p65 in KPC cells, scale bars: 10 µm. The intensity trace (offset white line) is plotted at the bottom. j) Co‐ip and western blot analysis of the endogenous DEF/p65 proteins interaction in the PANC1 and HPAC. k) Co‐ip and western blot analysis of the exogenous DEF/p65 proteins interaction in the 293T cells co‐transfected with Flag‐tagged DEF and His‐tagged p65. l) Stability analysis of p65 in control and DEF knockdown PANC1 cells treated with CHX (300 µm) for the indicated time. The representative image is shown. m) Ubiquitination assay of p65 in control and DEF KD PANC1 cells, subjected to anti‐p65 IP and anti‐ubiquitin western blot analysis after treatment with MG132(10 µM, 24 h). All the data were calculated by unpaired two‐tailed Students’*t*‐tests. ns, not significant, ^**^ *p* < 0.01, ^***^ *p* < 0.001. RT‐qPCR, real‐time quantitative polymerase chain reaction. Representative data from triplicate experiments are shown, and error bars represent SEM. All data are representative of three independently performed experiments.

Previous studies have demonstrated that the depletion of DEF results in the accumulation of p53 protein, suggesting that DEF mediates p53 degradation through the cysteine protease calpain3, independent of the proteasome pathway.^[^ ^17^ ^]^ Analysis of TCGA database revealed a significant correlation between DEF and p53 expression (Pearson's r = 0.75, *p* < 0.001), as well as between p53 and calpain3 expression (Pearson's r = 0.35, *p* < 0.001) in normal pancreas tissue (Figure S2a, Supporting Information). However, we observed no significant alteration in p53 mRNA or protein expression level upon DEF knockdown in pancreatic cancer cells (**Figure** **4**a,b). Also, p53 expression was not considerably affected by DEF overexpression in pancreatic cancer cells (Figure S2b, Supporting Information). In addition, no correlation was observed between DEF and p53 expression (Pearson's r = 0.24, *p* = 0.0012) or between p53 and calpain3 expression (Person's r = 0.11, *p* = 0.12) in pancreatic cancers (Figure S2c, Supporting Information). These results suggest that DEF may play distinct regulatory roles in pancreatic cancer independent of its interaction with p53.

To investigate the underlying molecular mechanisms by which DEF modulates pancreatic cancer progression, we performed RNA sequencing on PANC1 cells to identify transcriptomic changes following DEF KD (Figure 4c). Heatmaps depicting differentially expressed genes between the control and DEF KD groups are presented in Figure 4d. According to Kyoto Encyclopedia of Genes and Genomes (KEGG) pathway analysis, a significant downregulation of the NF‐κB signaling pathway was observed (Figure 4e).^[^ ^28^ ^]^ The NF‐κB signaling pathway is activated in response to stimuli such as cytokines and pathogens. Upon activation, the IKK complex phosphorylates IκBα, leading to its degradation and subsequent release of NF‐κB(p65/p50). NF‐κB then translocates to the nucleus where it binds to specific DNA sequences and initiates the transcription of target genes (Figure 4f).^[^ ^22^ ^]^ To elucidate the potential involvement of DEF in regulating the NF‐κB signaling pathway, we assessed the protein expression levels of key factors (Figure S2d, Supporting Information). The knockdown of DEF resulted in the suppression of the mRNA expression levels of the cytokines *IL‐1β*, *IL‐6*, and *IL‐8* (Figure S2e, Supporting Information). Similarly silencing DEF in PDAC cells significantly reduced the mRNA levels of *TNFAIP3*, *TRAF2*, and *C‐IAP2* (Figure S2f,g, Supporting Information), as well as the protein expression levels of BCL2 and C‐IAP1(Figure S2h, Supporting Information). Therefore, our findings imply that the role of DEF in PDAC is likely intricately associated with the NF‐κB signaling pathway.

Considering that p65 plays a crucial role as a transcription factor in the NF‐κB signaling pathway, we assessed the clinical significance of p65 in pancreatic cancer. Similar to DEF, higher expression of p65 was observed in tumor samples compared to paired normal tissues (Figure S3a,b, Supporting Information). Depletion of p65 induced apoptosis and inhibited cancer cell growth, thereby highlighting a strong correlation between p65 and the prognosis of pancreatic cancer patients (Figure S3c–n, Supporting Information). Consequently, we hypothesized that DEF and p65 were potentially associated. To explore this hypothesis, we evaluated the mRNA levels of p65 after DEF knockdown in pancreatic cancer cells and observed that the transcription level of p65 remained unchanged (Figure S4a, Supporting Information). We then examined protein expression levels of p65 after DEF KD. The results demonstrated that DEF KD cells exhibited reduced abundance of both p65(Figure 4g; Figure S4b, Supporting Information) and phosphorylated p65 (p‐p65) (Figure 4g). Given that p65 is an important mediator in tumor progression, we further explored the regulatory effects of DEF on p65 expression. Positive correlations were consistently observed across multiple experimental assays including protein analysis in paired clinical tissue samples (Figure S4c, Supporting Information) and tumor tissue microarrays (Figure 4h; Figure S4d, Supporting Information). These discoveries provide additional support for the significance of DEF in this context. Moreover, immunofluorescence analysis revealed a diffuse cytoplasmic distribution of DEF in pancreatic cancer cells, which co‐localized with p65 (Figure 4i; Figure S3f, Supporting Information). To further investigate the relationship within the cytoplasm, we conducted co‐immunoprecipitation (Co‐ip) experiments. The results of these experiments suggested an endogenous interaction between DEF and p65 in pancreatic cancer cells (Figure 4j; Figure S4e, Supporting Information) as well as an exogenous interaction in 293T cells (Figure 4k). We treated tumor cells with cycloheximide to investigate the degradation of p65 protein following DEF KD, revealing that DEF KD induces accelerated degradation of p65 (Figure 4l). Previous studies have demonstrated that the degradation of p65 is mediated by ubiquitination^[^ ^29^, ^30^ ^]^; therefore, we examined this process in pancreatic cancer cells with DEF KD. Our results indicate that DEF is involved in the inhibition of ubiquitination‐mediated p65 degradation (Figure 4m). Collectively, these findings suggest that DEF impedes ubiquitination‐mediated proteasomal degradation of p65, thereby facilitating tumor progression in pancreatic cancer.

### 2.4 DEF Specifically Interacts with p65 through Two Motifs

**Figure 5:**
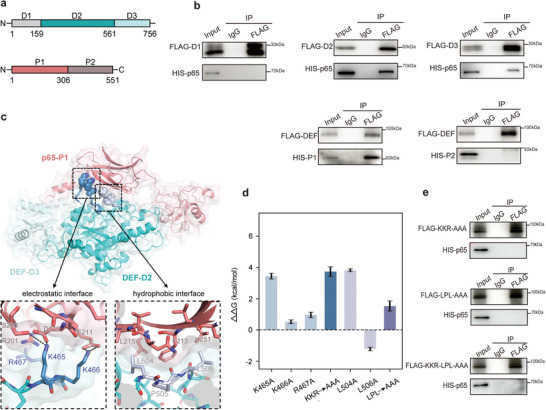
DEF residues K465‐K466‐K467 and L504‐P505‐L506 are crucial for the specific binding of the p65 protein. a) Schematic illustration of the structure of DEF and p65. b) Co‐ip and western blot analysis of the interaction between Flag‐tagged DEF mutants and His‐tagged p65 in 293T cells (at the top). Co‐ip and western blot analysis of the interaction between His‐tagged p65 mutants and Flag‐tagged DEF in 293T cells (at the bottom). c) Predicted model of the complex of DEF (cyan) and p65 (deep salmon). The negatively charged (sky blue) and hydrophobic (light purple) residues from two loops are mainly involved in the composition of the complex interface, represented as spheres and shown in the panels below with enlarged views. d) Relative binding free energy difference (ΔΔG) of mutations of interacting residues on DEF. Both single mutations (light blue) and triple mutations (dark blue) results were calculated using the free energy perturbation (FEP) (see Table S2, Supporting Information, for detailed values). e) Co‐ip and western blot analysis of the interaction between His‐tagged p65 and Flag‐tagged DEF mutants (KKR, K465A, K466A, and R467A; LPL, L504A, P505A, and L506A; K‐L, K465A, K466A, and R467A, L504A, P505A, and L506A) in the 293T cells. Co‐ip analyses were performed with the indicated antibodies. Statistical analysis of three independent experiments is performed.

Subsequently, we employed computational modeling and in vitro experiments to investigate the intricate binding mechanism of this pivotal interaction. Initially, a series of DEF and p65 truncations were generated to evaluate their respective binding capabilities. Based on the structure model predicted by Alphafold,^[^ ^31^, ^32^ ^]^ the full‐length DEF was rationally divided into three domains: DEF domain 1 (D1, residue 1–159), DEF domain 2 (D2, residue 160–561), and DEF domain 3 (D3, residue 562–756) (**Figure** **5**a). Similarly, p65 was partitioned into two domains: p65 domain 1 (P1, residue 1–306) and p65 domain 2 (P2, residue 307–551) (Figure 5a). Co‐immunoprecipitation analysis revealed that the crucial protein‐protein interaction primarily involved individual D2 and D3 of DEF along with P1 of p65 (Figure 5b). Subsequently, the structure models for the DEF/p65 complex were constructed using AlphaFold artificial intelligence platform (Figure S5a, Supporting Information, for predicted complexes of D2/P1, D3/P1 and D2‐D3/P1), which were further refined through molecular dynamics (MD) simulations following established protocols from our previous studies on protein dynamics^[^ ^31^, ^32^, ^33^, ^34^, ^35^ ^]^ (results shown in Figure S5b,c, Supporting Information). A representative snapshot from MD simulations of the selected D2‐D3/P1 complex is depicted in Figure 5c. Dynamic structural analysis (Figure S5d, Supporting Information) and in silico mutagenesis (free energy perturbation calculations for alanine substitutions) revealed that two key binding motifs on DEF, K465‐K466‐R467 (KKR) and L504‐P505‐L506 (LPL), contributed significantly to this interaction (Figure 5d; refer to Table S2, Supporting Information, for detailed values). Finally, the alanine mutants were generated for both motifs (KKR→AAA and LPL→AAA), and their essential roles in mediating DEF/p65 interaction were validated using Co‐ip analysis (Figure 5e). These findings strongly support that DEF indeed interacts with p65 via KKR (K465‐K466‐R467) and LPL (L504‐P505‐L506) motifs to fulfill its critical functional role.

### 2.5 Peptide Designed to Mimic DEF Down‐Regulates p65 Expression and Exerts Anti‐Tumor Effects

**Figure 6:**
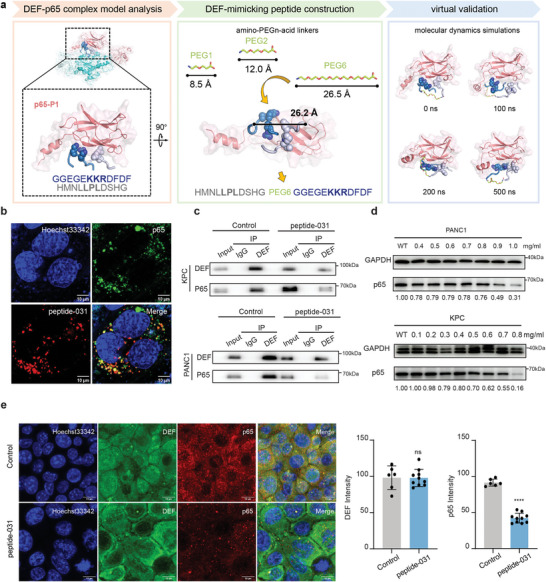
DEF‐mimicking peptide that specifically reduces p65 expression was designed. a) Computational virtual design flow of DEF‐mimic peptides targeting p65. First, the two DEF loops with key interacting residues are selected, and the end‐to‐end distances are measured. Subsequently, amino‐PEGn‐acid linkers are selected to fit the end‐to‐end distances. Finally, molecular dynamics simulations validate the binding between the PEGn‐linked peptide and p65. b) Representative images showing co‐localization of peptide‐031 (Red) and p65 (Green) in KPC cells with p65 overexpression treated with indicates peptides at 0.8 mg mL^−1^ for 24 h. Scale bars: 10 µm. c) Co‐ip assays of DEF‐p65 interaction in peptide‐031 treated PANC1 (0.9 mg mL^−1^, 24 h) and KPC cells (0.8 mg mL^−1^, 24 h). d) Western blot analysis of p65 of PANC1 and KPC cells treated with the indicated peptides at various concentrations for 24 h. e) Immunofluorescence staining and statistical analysis of DEF and p65 in KPC cells treated with the indicated peptides, scale bars: 10 µm. Quantification of DEF and p65 fluorescence intensity are plotted at right. Unpaired two‐tailed Student's *t*‐tests were used on all the data. ns, not significant, ^****^ *p* < 0.0001. Representative data from triplicate experiments are shown, and error bars represent SEM. All data are representative of three independently performed experiments.

Given the absence of available DEF inhibitors, the *de novo* design of a DEF‐mimicking peptide that disrupts the DEF‐p65 interaction presents a promising approach toward further validating the function of DEF. Based on the aforementioned binding interactions between DEF and p65, we propose a computational workflow for designing these peptides, which encompasses the following steps (**Figure** **6**a): i) determination of two fragments of DEF containing key interacting motifs (GGEGE **KKR** DFDF and HMNL **LPL** DSHG); ii) utilization of amino‐PEGn‐acid linkers (where PEG represents polyethylene glycol) to connect these fragments with PEG linker length tailored to suit their end‐to‐end distances; iii) subsequent MD simulations performed to validate the binding capabilities of PEGn‐linked peptides with p65 (Figure S5e, Supporting Information); and finally, iv) construction and synthesis of the desired peptide, namely peptide‐031 (HMNLLPLDSHG‐PEG6‐GGEGEKKRDFDF), as a potential therapeutic candidate for interfering with DEF‐p65 interactions and exerting anti‐tumor effects.

To assess the specificity of peptide‐031 toward p65 expression in pancreatic cancer cells, we conducted live imaging experiments, which revealed that peptide‐031 exhibited remarkable intracellular infiltration and demonstrated prominent co‐localization with p65 (Figure 6b). Moreover, our findings indicated that peptide‐031 effectively attenuated the interaction between DEF and p65 (Figure 6c). Increasing concentrations of peptide‐031 during cell incubation resulted in a notable reduction in p65 expression, illustrating the dose‐dependent effect of peptide‐031 on the suppression of p65 (Figure 6d) and phosphorylated p65 (Figure S6a, Supporting Information). Additionally, immunofluorescence staining revealed a remarkable reduction in the fluorescence intensity of p65 after incubation with peptide‐031 while maintaining unchanged fluorescence intensity for DEF (Figure 6e). These results underscore the high specificity of peptide‐031 in targeting p65.

**Figure 7:**
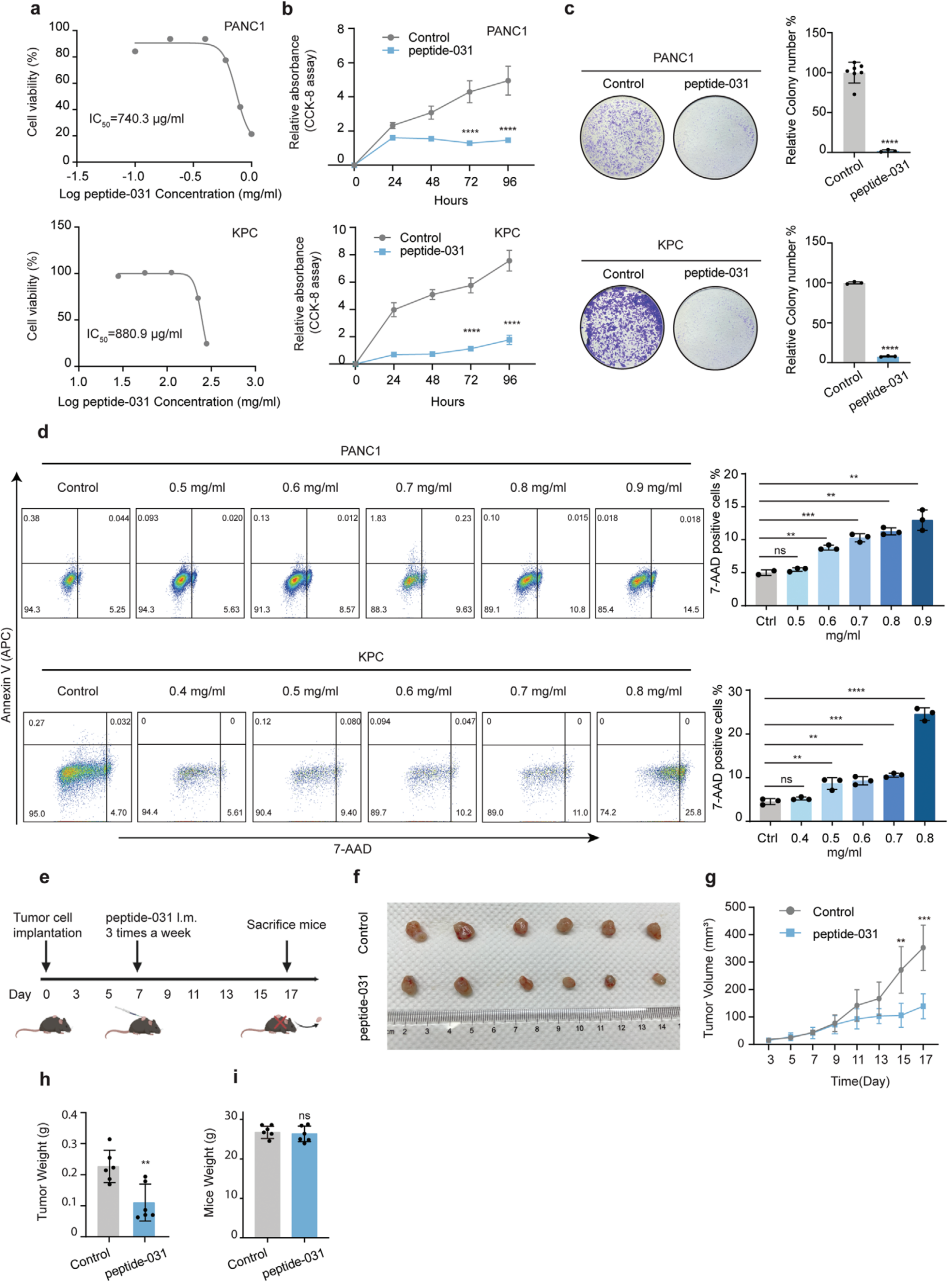
DEF‐mimicking peptide effectively exerts anti‐tumor effects. a) Determination of IC_50_ values of peptide‐031 for PANC1 (top) and KPC (bottom) at increasing inhibitor concentrations for 24 h. b) The cell viability of peptide‐031 treated in pancreatic cancer cells by CCK‐8 assays. c) Representative images and quantitation of colony formation after peptide‐031 treatment in PANC1 (top) and KPC cells (bottom). d) Flow cytometry analysis and quantification of 7‐AAD+ pancreatic cancer cells treated with peptide‐031 at various concentrations for 24 h; *n* = 3 biologically independent experiments. e) Schematic protocol of the combination of peptide‐031 therapy followed for up to 17 days of xenograft tumors in nude mice. f) Tumor growth curve of mice treated with peptide‐031 (500 µg/mouse) and PBS therapy in xenograft models. g) Representative images of tumors (*n* = 6) and their weights h) were measured in the indicated groups. Tumors were measured at specified time points and dissected at the endpoints (*n* = 6). i) The body weight of mice was measured in the indicated groups. Unpaired two‐tailed Student's *t*‐tests were used on all the data. ns, not significant, ^**^ *p* < 0.01, ^***^ *p* < 0.001, ^****^ *p* < 0.0001. Representative data from triplicate experiments are shown, and error bars represent SEM.

Subsequently, we evaluated the effects of peptide‐031 on pancreatic cancer cells. The IC_50_ value of peptide‐031 was determined to be 740.3 µg mL^−1^ for PANC1 and 880.9 µg ml^−1^ for KPC (**Figure** **7**a). Treatment with peptide‐031 resulted in a significant inhibition of tumor growth (Figure 7b,c), and an increase in the number of dead cells (Figure 7d). In addition, treatment with peptide‐031 led to a significant reduction in the expression of *IL‐1β* and *TNFα* (Figure S6b, Supporting Information). To assess the impact of peptide‐031 on tumor growth in vivo, we administered it via intratumoral injection to KPC‐bearing mice (Figure 7e). Our results showed a substantial reduction in both tumor volume (Figure 7f,g) and weight (Figure 7h). However, no difference was observed in the body weights of mice between the two groups (Figure 7i). We evaluated the safety of the peptide‐031 in mice by examining the liver, lung, heart, and kidney tissues with H&E staining post‐treatment, the findings indicate no adverse morphological changes, implying that the peptide‐031 exhibits a favorable safety profile (Figure S6c, Supporting Information). These results suggest that developing inhibitors to block the binding between DEF and p65 may present a potential therapeutic alternative for treating pancreatic cancer.

## 3 Discussion

Over the past decade, extensive scientific investigations have illuminated the fundamental role of DEF in driving the expansion and development of digestive organs.^[^ ^16^, ^17^, ^36^ ^]^ However, the comprehensive elucidation of DEF's functions beyond its established role in digestive organ development remains underexplored. Consequently, further investigations are necessary to unravel the complex molecular mechanisms by which DEF modulates diverse biological processes. In this study, we found that DEF acts as a pro‐tumor factor in pancreatic cancer. We observed elevated cytoplasmic expression of DEF in pancreatic cancer cells and demonstrated its ability to promote tumor proliferation through interaction with p65. Utilizing virtual computational screening methods, we successfully developed peptide‐031 to specifically disrupt the DEF‐p65 interaction, thereby inhibiting cancer growth both in vitro and in vivo. Our study not only identifies a novel target for pancreatic cancer treatment but also presents peptide‐031 as a promising peptide‐based inhibitor worth further investigation.

Previous reports have indicated that DEF is predominantly localized in the nucleus and plays a crucial role in ribosome biogenesis and facilitating calpain3‐mediated degradation of p53 protein during digestive organ development.^[^ ^17^ ^]^ Intriguingly, our study reveals significant cytoplasmic localization of DEF in pancreatic cancer cells, which is associated with its ability to promote tumor growth by stabilizing p65 independently of p53. This finding highlights the diverse biological roles of DEF under different contexts and environments,^[^ ^36^ ^]^ particularly within the realm of pancreatic cancer. Therefore, the subcellular distribution of DEF within pancreatic cancer cells may significantly influence its functional properties: nuclear DEF mediates the degradation of p53,^[^ ^22^ ^]^ however, cytoplasmic DEF exhibits a distinct function that is independent of p53 in malignant cells. Similarly, an increased cytoplasmic‐to‐nuclear ratio of UTP18 has been observed during normal‐adenoma‐carcinoma progression in colorectal tissues. Cytoplasmic UTP18 has been shown to regulate cell cycle progression through mediating the degradation of tumor suppressor p21.^[^ ^37^ ^]^ These findings indicate that cytoplasmic localization of UTP family members might be common in tumor cells. However, the underlying regulatory mechanism responsible for this shift remains unclear and could potentially involve post‐translational modifications such as deSUMOylatio‐mediated processes.^[^ ^37^ ^]^ Further investigations into the dynamic nucleocytoplasmic localization mechanisms governing UTP family members are warranted to gain comprehensive insights into their distinct roles in tumorigenesis and to reinforce their potential as therapeutic targets in cancer treatment.

To date, no specific anti‐tumor medication exploiting the binding mechanism of DEF to p65 has been developed. Peptides, polymers ranging from 5 to 40 amino acids in length, have emerged as a crucial area of investigation in both biological and medical studiese.^[^ ^38^ ^]^ Their compact size, high specificity and selectivity, coupled with low immunogenicity, make peptides attractive as potential cancer therapeutics.^[^ ^39^, ^40^ ^]^ In this study, we employed in silico methods including AlphaFold artificial intelligence platform and protein dynamics simulations to predict the structure of DEF and its bound state with p65. Based on the predicted complex model, we identified and validated two key binding motifs on DEF that contribute predominantly to its interaction with p65. Furthermore, leveraging a computational‐assisted design workflow, we develop a DEF‐mimicking peptide, named peptide‐031, which specifically target p65. This synthetic inhibitor exerts a potent anti‐tumor effect by mimicking the key properties of DEF and competitively binding to p65 while preserving DEF's other functions intact. These results illustrate that DEF promotes cancer progression in pancreatic cancer, primarily by interacting with p65. Therefore, targeting the interaction between DEF and p65 could be a promising innovative strategy for the development of targeted therapies for pancreatic cancer.

However, the clinical applications of peptide‐031 are limited. This DEF‐mimicking inhibitor we constructed has limited binding affinity to p65, likely due to the limited binding affinity of the DEF‐p65 interaction. Therefore, peptide‐031 can be modified to enhance its binding capacity to p65, resulting in more effective inhibition of DEF‐p65 interactions. Notably, p65 is constitutively expressed in various types of normal cells^[^ ^41^ ^]^; therefore, targeted inhibitors or peptides against p65 must possess sufficient specificity and selectivity to ensure their exclusive impact on p65 in tumor cells while sparing normal cells.^[^ ^42^, ^43^ ^]^ Consequently, further research is warranted to assess the efficacy, safety profile, tolerability, and potential side effects of peptide‐031. Furthermore, pancreatic cancer cells are enveloped by dense stroma, which impedes the penetration of peptides into tumors and hampers their access to cancerous cells.^[^ ^44^ ^]^ Henceforth, it will be important to enhance the selectivity of peptides toward pancreatic cancer by conjugation with specific peptide sequences to augment binding affinity, or by utilizing nanotechnology or liposomes for targeted delivery, thus ensuring specificity to the tumor cells. Furthermore, comprehensive investigations are necessary for optimizing the in vivo performance of peptide‐031.

In conclusion, our findings demonstrate that DEF serves as a prognostic factor in pancreatic cancer. DEF interacts with and stabilizes p65, protecting it from ubiquitination‐mediated degradation, thereby promoting tumor proliferation in pancreatic cancer. Subsequently, by elucidating the underlying binding mechanism, a specific peptide‐031 was successfully developed to disrupt the formation of the DEF‐p65 complex. Remarkably, peptide‐031 exerts promising anti‐tumor effects both in pancreatic cancer cells and mouse models. Consequently, our clinical analyses establish the prognostic significance of DEF in pancreatic cancer while highlighting the potential therapeutic value of both DEF and peptide‐031 for further preclinical studies of pancreatic cancer treatment.

## 4 Experimental Section

### Cell Culture and Cell Transfection

The KPC cells were generated from spontaneous tumors in the Kras^LSL‐G12D^;Trp53^LSL‐R172H^; Pdx1‐Cre mouse model, which was a kind gift from the laboratory of Prof. Raghu Kalluri (MD Anderson Cancer Center, Houston, TX, USA). KPC and Capan2 cells were cultured in modified McCoy's 5A Medium (Thermo Fisher Scientific, Waltham, MA, USA) with 10% fetal bovine serum (FBS, Gibco), 100 U mL^−1^ penicillin and 100 µg mL^−1^ streptomycin (Genom, Hangzhou, China). PANC1 and HEK293T cells were cultured in high glucose Dulbecco's Modified Eagle Medium (DMEM) (Gibco, Grand Island, NY, USA) with 10% fetal bovine serum and 100 U mL^−1^ penicillin‐streptomycin. HPAC cells were cultured in Advanced DMEM/F‐12 (Thermo Fisher Scientific, Waltham, MA, USA) with 5% FBS, 100 U mL^−1^ penicillin‐streptomycin, 0.002 mg mL^−1^ insulin (Selleck, S6955), 0.005 mg mL^−1^ transferrin (MedChemExpress, #HY‐P70620),40 ng mL^−1^ hydrocortisone (MedChemExpress, #HY‐N0583), and 10 ng mL^−1^ mouse epidermal growth factor (MedChemExpress, #HY‐P1960). The PANC1, HPAC, Capan2, and HEK193T cell lines were obtained from the American Type Culture Collection (Manassas, VA, USA). All cells were incubated at 37 °C in a humidified chamber containing 5% CO_2_. The presence of mycoplasma contamination was routinely evaluated in all cells using PCR.

### Western Blot and Co‐Immunoprecipitation (Co‐IP)

Cells were washed with PBS and lysed using RIPA lysis buffer (Beyotime Biotechnology, #P0013B) containing phenylmethanesulfonyl fluoride (Beyotime Biotechnology, #ST505) and protease inhibitors (Beyotime Biotechnology, #P1005) for 30 min on ice. After that, the mixture was centrifuged at 12000 r.p.m. for 15 min. For nuclear and cytoplasmic extraction, NE‐PER Nuclear and Cytoplasmic Extraction Reagents kit (Thermo Fisher Scientific, #78 833) was used according to the manufacturer's instructions. The protein concentrations were measured by a bicinchoninic acid (BCA) reagent (Beyotime Biotechnology, #P0012). Protein samples were boiled to 100 °C in NuPAGE LDS sample buffer (4x) (Thermo Fisher Scientific, #NP0007) for 3–5 min. The lysates are separated by sodium dodecyl sulfate‐polyacrylamide gel electrophoresis and transferred to polyvinylidene fluoride membranes (Millipore, MA, USA). The membranes were incubated with the indicated antibody at 4 °C overnight after blocking with 5% milk in TBST for 2 h at room temperature. After washing with TBST thrice, the membranes were incubated with species‐specific primary secondary antibodies for 2 h at room temperature. The signals from immunoreactive proteins were detected using an Immobilon Western Chemiluminescent HRP Substrate (Millipore, #WBKLS0500). For the peptide treatment, PANC1 and KPC cells were treated with the indicated concentrations for 24 h, then conducted the above procedures. The correspondent bands were imaged using ChemiScopeTouch (Clinx Science Instruments, Shanghai, China). Three replicates were performed for all western blot analysis.

For co‐immunoprecipitation (IP) experiments, cells were lysed in IP/western lysing solution (50 mm Tris‐HCl pH 7.6, 320 mm NaCl, 0.1 mm EDTA, 0.5% NP‐40) including a phosphatase inhibitor cocktail (Bimake, #B15001) and a protease inhibitor cocktail (Bimake, #B14001) for 60 min on ice, then centrifuged 12000 r.p.m. for 20 min. The soluble protein supernatant was collected and incubated with indicated primary antibodies overnight at 4 °C. Then antibody‐protein complexes were precipitated with protein A/G beads (Bimake, #B23201) for 4 h at 4 °C. The protein beads were heated to 100 °C in NuPAGE LDS sample buffer(4x) for 3–5 min, then immunoprecipitation and western blot assays were described above. For the peptide treatment, PANC1 (0.9 mg mL^−1^, 24 h) and KPC cells (0.8 mg mL^−1^, 24 h) were treated with the indicated concentrations for 24 h, then conducted the above procedures. The intensity of immunoblot bands was evaluated using Image J 1.53t software (National Institutes of Health, Bethesda, USA).

### Generation of DEF/p65 Knockdown Cells

Two DEF‐specific shRNAs and two p65‐specific shRNAs were designed (sequences listed in Table S4, Supporting Information), and a control nonsense sequence was used as a negative control (Oobio, Shanghai, China). 7.5 µg lentiviral plasmid, 5.625 µg pdPAX2, and 1.875 µg pMD2.G were transfected into HEK293T cells in 10 cm plates by polyethylenimine (Polysciences, #24765‐1) according to the manufacturer's instructions. After 24 h, the fresh medium was replaced. Lentivirus‐containing media were harvested and filtered through a 0.45 µm filter after 48 h. Then target cells were infected with lentiviruses and replaced with fresh medium after 10 h. After 72 h, the transfected cells were selected by 10 µg mL^−1^ puromycin (InvivoGen, San Diego, CA, USA) for one week for stable knockdown cells.

### RNA Extraction and Quantitative Real‐Time PCR

Total RNA was exacted from cells or tissues using FastPure Cell/Tissue Total RNA Isolation Kit V2 (Vazyme Biotech Co., Ltd). RNA concentration was measured by a Thermo Scientific NanoDrop One instrument. RNA was reverse transcribed into cDNA using a PrimeScript RT reagent kit (Takara, #RR047A) followed by the manufacturer's instructions. Quantitative real‐time PCR was conducted using a TB Green Prime Ex Taq II Kit (Takara, #RR820A) with an Applied Biosystems 7500 Fast Real‐Time PCR System. The relative transcript levels of the target genes were normalized to *GAPDH* and calculated by the standard 2^−△△Ct^ method. Sequence information for primers for quantitative real‐time PCR was listed in Table S3 (Supporting Information).

### Cell Growth Assay, Edu Assays, and Colony Formation Assay

Cells were cultured at 80% confluence in 10 cm plates, after washing twice with phosphate‐buffered saline (PBS), cells were digested and seeded into 24‐well plates for cell growth assay (7000 cells each well) and into 6‐well plates for colony formation assay (500 cells each well). For cell growth assay, at days 1, 2, 3, and 4, 1:20 dilution of Cell Counting Kit‐8 (GLPBIO, #GK10001) was added into each well, and the cells were incubated for 2 h while protected from light. Then the optical density was detected at OD450 nm with a microplate reader (Synergy Neo2, BioTek, USA). For the colony formation assay, after two weeks cultured, colonies were fixed with 4% paraformaldehyde (PFA) (Thermo Fisher Scientific, #28 908) for 30 min. After washing with PBS 3 times, the cells were stained with 0.1% crystal violet for 20 min. Visible colonies were counted and scanned. Each treatment group was measured in triplicate. For EdU assay, BeyoClick EdU Cell Proliferation Kit with Alexa Fluor 594 (Beyotime, #C0078S) was used to measure cell proliferation, following the manufacturer's instructions. After EdU staining, cells were imaged with a Leica Stellaris 8 confocal laser scanning microscope, and the percentage of EdU‐positive cells was calculated in the total cells.

### Immunohistochemical Analysis (IHC)

For immunohistochemical analysis, mouse tumors harvested at the end of the animal experiments were stored in 10% neutral buffered formalin, embedded in paraffin. Tissues were sliced into 4 µm thick sections, placed onto super frost+ glass slides, baked for 60–90 min at 68 °C, and then deparaffinized. Antigen‐retrieval was performed by incubation in citrate antigen retrieval solution (Solarbio Life Science, Beijing, China), and the sections were boiled for 10 min, followed that they were incubated at room temperature for 25–30 min. Samples were blocked by 3% BSA for 30 min at room temperature. Sections were incubated with indicated primary antibodies at 4 °C overnight, followed by staining using an HRP‐conjugated secondary antibody for 50 min at room temperature. The target protein was visualized using a diaminobenzidine (DAB) chromogen kit (BDB2004, Biocare Medical, Pacheco, USA), in which the brown staining represented the targeted molecule. Slides were counterstained with diluted hematoxylin for 3–5 min. Representative images per tumor were captured using ImageScope software (Leica Biosystems, Wetzlar, Germany). Immunohistochemical staining of paraffin‐embedded PDAC tissue microarray slides were performed by Wuhan Servicebio Technology (Wuhan, China). The staining images were quantified by processing the images using 3D HISTECH quant center 2.1 software (3DHISTECH Kft, Budapest, Hungary).

### Immunofluorescence Staining

KPC cells were seeded on cover slides in 4‐well Chamber slides and cultured for 20 h. For peptide treatment assays, PANC1 (0.9 mg mL^−1^, 24 h) and KPC cells (0.8 mg mL^−1^, 24 h) were treated with the indicated concentrations for 24 h. Afterward, cells were fixed with 4% paraformaldehyde (Sigma, #F8775) for 30 min, permeated by 0.2% Triton X‐100 (Sigma, #T8787) for 20 min, and blocked in 5% BSA (Sigma, #SRE0096) for 120 min at room temperature. Primary antibodies were diluted in 5% BSA and incubated overnight at 4 °C, then secondary antibodies were added for 2 h at 37 °C, respectively. Hoechst33342 (Thermo Fisher Scientific, #62 249) was stained at room temperature for 40 min before mounting. Finally, cells were observed using the Leica Stellaris 8 confocal laser scanning microscopy. Data were analyzed with ImageJ 1.53t software.

### Flow Cytometric Analysis

For the cell apoptosis assay, cells were evaluated using an APC Annexin V Apoptosis Detection Kit with PI (Biolegend, #640 932). Cells were digested from 24 wells and washed twice with the cold Biolegend's cell staining buffer, and subsequently incubated cells with 5 µL of Annexin V‐APC and 10 µL of Propidium Iodide solution for 20 min at room temperature in the dark. For the peptide treatment, PANC1 and KPC cells were treated with the indicated concentrations for 24 h, then conducted the above procedures. Finally, flow cytometry (Beckman CytoFlex, USA) evaluated the percentage of apoptosis cells and analyzed them using Flowjo Software (Flowjo, LLC).

### Human Subject

Human pancreatic adenocarcinoma cancer tissue and adjacent normal tissue specimens were obtained from the Department of Hepatobiliary and Pancreatic Surgery, the First Affiliated Hospital, School of Medicine, Zhejiang University. Paraffin‐embedded PDAC tissue array slides comprising 156 samples from patients were created by the Wuhan Servicebio technology using PDAC tissue specimens from the First Affiliated Hospital, School of Medicine, Zhejiang University. The tumor specimens contained the following clinical information: patient age, gender, CA19‐9, the tumor‐node‐metastasis (TNM) staging system, vascular invasion, lymphatic metastasis, and distant metastasis. Samples from this study were collected from patients with pancreatic cancer between 2012 and 2017. All patients were diagnosed with pancreatic cancer by histopathological examination respectively. Written informed consent was obtained from all patients at the time of enrollment. The protocol was approved by the Institutional Review Board at the First Affiliated Hospital, School of Medicine, Zhejiang University.

### Animal Studies

All animal studies were conducted according to the guidelines for the care and use of laboratory animals and were approved by the Institutional Biomedical Research Ethics Committee of the First Affiliated Hospital, School of Medicine, Zhejiang University. DEF knockdown and control human pancreatic cancer cells were re‐suspended in a density of 5 × 10^6^ cells in 50 µL serum‐free DMEM and separately injected subcutaneously into the right flank of 6‐8‐weeks‐old‐male nude mice. The incidence of tumors was recorded. Xenograft tumor growth was measured every 5–7 days up to 30 or 40 days. Tumor volume was recorded by the formula: tumor volume = (length x width^2^)/2. Tumor size and weights were recorded. Tumors were harvested for histopathological examination and further analysis. For the orthotopic models, a small incision was made in the left abdomen near the spleen of 6‐8‐weeks‐old‐male C57BL/6 mice, where the pancreas was located in front of the right side of the spleen. DEF knockdown and control KPC cells at a density of 5 × 10^5^ cells in 20 µL serum‐free McCoy's 5A mixed with 10 µL Matrigel were injected into the pancreas using a sterile insulin needle. Following the experiments described above. For the survival experiments, the time point of death of each mouse was tracked and recorded, and a survival curve was plotted. For peptide treatment assays, mice were randomly assigned into two treatment groups: PBS and peptide‐031 (*n* = 5 per group). Mice from the peptide‐031 groups were intratumorally injected with 500 µg/ peptide in PBS every two days. Mice from the PBS groups were intratumorally injected with PBS every two days.

### RNA‐Sequencing and Enrichment Analysis of Gene Network and Pathway

Transcriptomic sequencing was performed in DEF knockdown or control PANC1 cells. RNA extraction was performed using TRIzol reagent (Invitrogen, #15 596 026, USA) and underwent genome‐wide transcriptomic analysis using Genergy‐Bio (Shanghai, China). The number and integrity of RNA were assessed using FastQC and RseQC software. According to the protocol of Illumina (mRNA‐SEQ Sample Preparation Kit), the cut RNA fragment was reverse transcribed to generate the cDNA library after PCR amplification. Peer sequencing was performed on an Illumina HiSeq 2500 (Genergy‐Bio, Shanghai, China), according to manufacturers’ recommendations. The raw data was handled by Skewer and data quality was checked by FastQC (v0.11.2). Clean reads were aligned to the reference genome using STAR (2.5.3a). Differentially expressed genes (DEGs) were determined by DEGseq2 (v1.16.1). DEGs were selected with fold change > 1.5 and P‐value < 0.05. Downregulated DEGs were then used as input for the Kyoto Encyclopedia of Genes and Genomes (KEGG) pathway enrichment analysis.

### Detecting Action of Peptide‐031 in Cells for IC_50_ Testing

To investigate the influence of peptide‐031 on cell viability, the PANC1 and KPC cells were cultured at 80% confluence in 10 cm plates, after washing twice with phosphate‐buffered saline (PBS), cells were digested and seeded into 96‐well plates for cell growth assay (5000 cells each well). Cells were treated with peptide‐031 at indicated concentrations (0.1, 0.2, 0.4, 0.8, 1.0, and 1.2 mg mL^−1^) for 24 h, and resulted in a dose‐dependent inhibition of cell growth with control cells. For cell growth assay, after 24 h 1:20 dilution of Cell Counting Kit‐8(GLPBIO, #GK10001) was added into each well, and the cells were incubated for 2 h while protected from light. Then the optical density was detected at OD450 nm with a microplate reader (Synergy Neo2, BioTek, USA). Plot the drug concentration on the x‐axis and the assay signal of absorbance on the y‐axis. Fit the data to a dose‐response curve using appropriate software or algorithms. Determine the IC_50_ value as the peptide‐031 concentration that inhibits the assay signal by 50%. Perform statistical analysis to assess the significance of the IC_50_ value.

### Complex Modeling

To predict the binding mode of DEF to p65, DEF and p65 were truncated based on pull‐down assays to avoid interference with the predictions by disordered regions of the two proteins. DEF D2 (residue 160–561), D3 (residue 562–756), and D2‐D3 (residue 160–756) were thus chosen to predict the complex with p65 P1 (residue 1–306). Five complex models were generated using Alphafold‐multimer.^[^ ^30^, ^31^ ^]^ The top‐ranked models of D2/P1, D3/P1, and D2‐D3/P1 were compared to validate the reproducibility and reliability of the prediction (Figure S5a, Supporting Information). The protein models were visualized using PyMOL.^[^ ^45^ ^]^

### Molecular dynamics simulations

Molecular dynamics (MD) simulations were used to verify the stability of D2‐D3/P1 model. The size of the solution system for the D2‐D3/P1 complex was 17 × 11 × 9 nm.^[^ ^45^ ^]^ All systems were solvated with TIP3P water.^[^ ^46^ ^]^ Na^+^ and Cl^−^ ions were added to the bulk water at a salt (NaCl) concentration of 150 mm. The final system sizes are nearly 150 000 atoms. All systems were run using GROMACS 2020.6^[^ ^47^ ^]^ for 500 ns in triplicate. The CHARMM36 force field for proteins was used.^[^ ^48^ ^]^ The temperature was maintained at 310 K and the pressure at 1 atm using V‐rescale^[^ ^49^ ^]^ and Parrinello‐Rahman,^[^ ^50^ ^]^ respectively. The cutoff distance of Van der Waals interactions was 12 Å. The long‐range electrostatic interactions were treated using the particle mesh Ewald method.^[^ ^51^ ^]^ The covalent bonds with hydrogen atoms were constrained by the LINCS algorithm,^[^ ^52^ ^]^ which allows a time step of 2 fs. All trajectory analyses (RMSD and distance, see Figure S5c,d, Supporting Information) were performed with VMD.^[^ ^53^ ^]^

### The Estimation of Differences in Binding Affinity Due to Mutations

The relative binding free energy differences (ΔΔGs) of alanine mutations were calculated by Free Energy Perturbation,^[^ ^54^, ^55^, ^56^ ^]^ and combined with Hamiltonian Replica‐Exchange Molecular Dynamics (FEP/HREX)^[^ ^57^ ^]^ for efficient convergence. The free energy changes for residue mutations were estimated in both the bound state (DEF and p65 complex) ΔG_bound_ and the free state (isolated DEF) ΔG_free_ using Gromacs 2020.6. Thus, the binding free energy change caused by residue mutation is estimated as ΔΔG = ΔG_bound_ − ΔG_free_. PMX^[^ ^58^, ^59^ ^]^ was used for preparing the dual‐topology files with CHARMM36 force field. For each mutation, 24 windows of sequential annihilation of electrostatics and van der Waals were set up and started from the same equilibrated system. Every mutation calculation was performed for at least 62 ns (1.3 ns × 24 windows  ×  2 states). The soft‐core potentials^[^ ^60^ ^]^ (α, the power for lambda term, the power of the radial term, and sigma were set to 0.5, 1, 6, and 0.3, respectively) were used during simulations. The exchange between neighboring windows was attempted every 1 ps. Hamiltonians of the systems were saved every 0.2 ps. ΔΔGs and their statistical errors were estimated from the last 1 ns simulation of each window using the Multistate Bennett Acceptance Ratio (MBAR) method^[^ ^61^ ^]^ in Alchemical Analysis.^[^ ^62^ ^]^

### Design Flow of DEF‐Mimicking Peptides

In order to design DEF‐mimicking peptides targeting p65, a computer virtual design was performed using the following process. First, two peptides were selected, GGEGEKKRDFDF (DEF residues 460–471) and HMNLLPLDSHG (DEF residues 500–510), which consist of 4–5 amino acid residues before and after the DEF's negatively charged (KKR) and hydrophobic (LPL) motif. Then, the distance was measured between the N‐terminus of one peptide and the C‐terminus of the other in the D2‐D3/P1 model. Next, the amino‐poly (ethylene glycol)‐acid (amino‐PEGn‐acid) of appropriate length was chosen as a linker to connect the two peptide termini and obtained various PEGn‐linked peptides. Finally, the binding of the PEGn‐linked peptides to p65 was verified by MD simulations. The structural models and force fields of the amino‐PEGn‐acid residues were generated by the CHARMM‐GUI^[^ ^63^ ^]^ module *Ligand Reader & Modeler*.^[^ ^64^ ^]^ The models of the PEGn‐linked peptide were built manually by PyMOL and equilibrated by MD simulations using GROMACS with the C‐α positional restraints. The complex models of PEGn‐linked peptide/p65 were built by aligning the portion of the amino acids of the peptide to the D2‐D3/P1 model. The solution system was built referring to D2‐D3/P1 and run for 500 ns to analyze binding stability (Figure S5e, Supporting Information). All peptides‐031 and cy5.5‐labeled peptides‐031 were chemically synthesized by CPC Scientific, with purity above 98%. All peptides were diluted with H_2_O at room temperature.

### Statistical Analysis

A Mann–Whitney U test or a two‐tailed t‐test was used to analyze the differences between the two groups. For multiple groups, one‐way or two‐way analysis of variance (ANOVA) with appropriate posttests was used. Two‐tailed tests were performed for all statistical analyses. Spearman's rank correlation was performed to analyze the correlation between variables. The log‐rank test was used to evaluate Kaplan–Meier survival curves. *P* values < 0.05 were considered significant. Adjusted *P* value metrics are stated at the end of each figure legend where applicable. The experiments were repeated independently with at least three biological replicates with similar results to demonstrate reproducibility. Microsoft Excel 2023, SPSS (Version 25, USA), and GraphPad Prism (Version 8.0, USA) software were performed to analyze all the data.

### Ethics Statement

The manuscript was written according to established ethical standards. The ethical standards of the Ethics Committee of the First Affiliated Hospital of Zhejiang University School of Medicine approved the experimental protocol. The animal experiments were performed following the applicable guidelines of the Animal Ethics Committee of the First Affiliated Hospital of Zhejiang University School of Medicine and the China Animal Protection Law.

## Conflict of Interest

The authors declare no conflict of interest.

## Author contributions

S.H., J.Y., and T.X. contributed equally to this work. X.B., T.L., R.Z., and J.Y. conceived and designed the research; S.H. performed most of the molecular and biochemical experiments, with assistance from J.Y., D.B., Y.H., and X.L.. J.Y. collected the clinical samples and information of patients with pancreatic cancer. Xie T and Jiang Y performed computer simulation. S.H., J.Y., Y.H., and X.L. performed xenograft experiments, IHC analysis, and CyTOF analysis. X.H. performed immunofluorescence imaging. X.B., T.L., R.Z., and D.Z. contributed to the data discussion. X.B., T.L., and R.Z. supervised the study. S.H., J.Y., and T.X. drafted the manuscript. X.B., T.L., and R.Z. revised and all authors approved the final version of the manuscript.

## Supporting information

Supporting Information

## Data Availability

All data are available via direct request from the corresponding author.
