# Correction to “Inhibition of DEF‐p65 Interactions as a Potential Avenue to Suppress Tumor Growth in Pancreatic Cancer”

**DOI:** 10.1002/advs.202505440

**Published:** 2025-07-14

*Adv Sci*. 2024 Jul;11(28):e2401845.

https://doi.org/10.1002/advs.202401845

We would like to address a few corrections related to the images published in our paper.

Figure 3a Correction: In the original published paper, the expression of GAPDH in DEF knockdown PANC1 cells was mistakenly chosen during the image layout. The corrected figure is provided below.

Corrected Figure 3a:


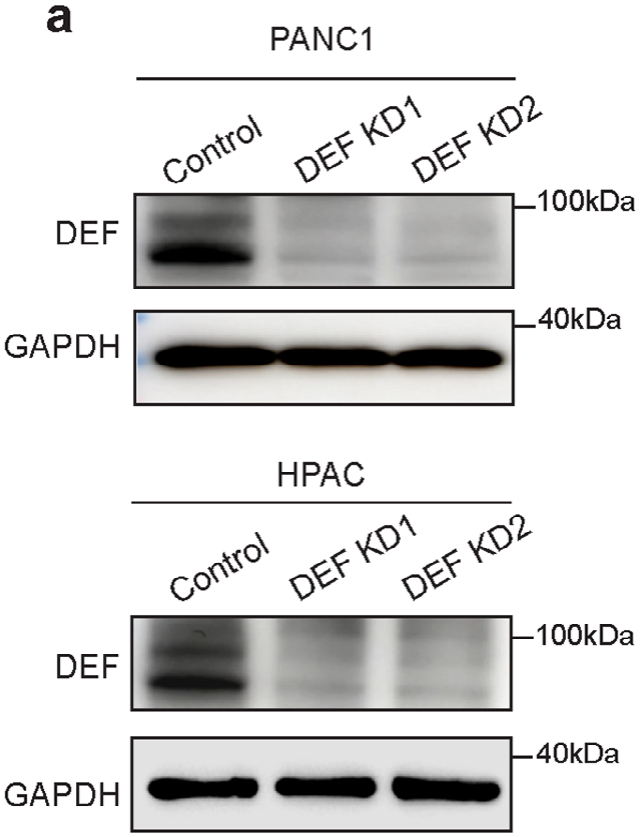


Figure 4j Correction: The expression of the p65 protein in PANC1 cells was mistakenly duplicated from the HPAC group in the original figure. The corrected figure is shown below.

Corrected Figure 4j:


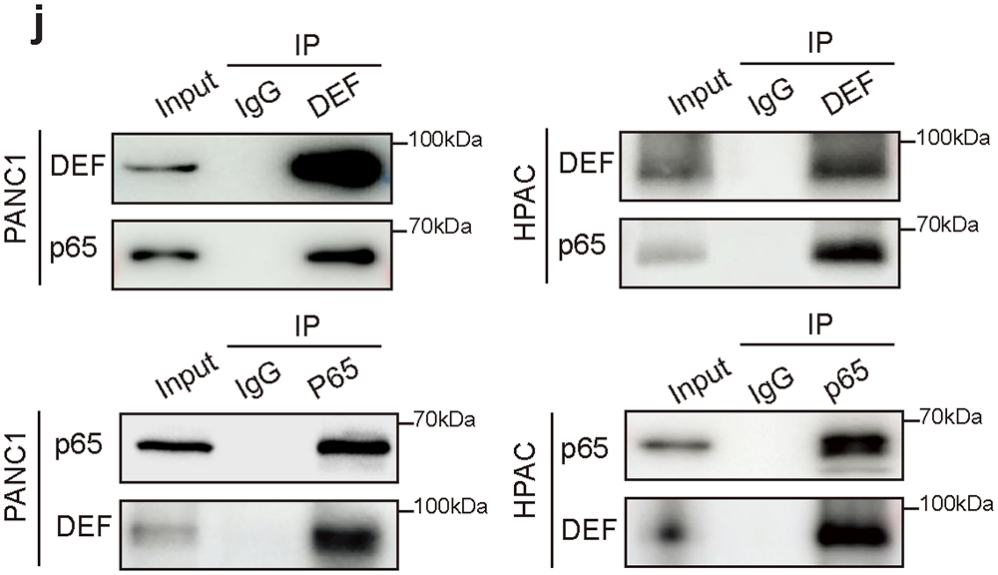


Figure 7c Correction: The colony formation after peptide‐031 treatment in PANC1 was mistakenly duplicated from the KPC group. The corrected figure is shown below.

Corrected Figure 7c:


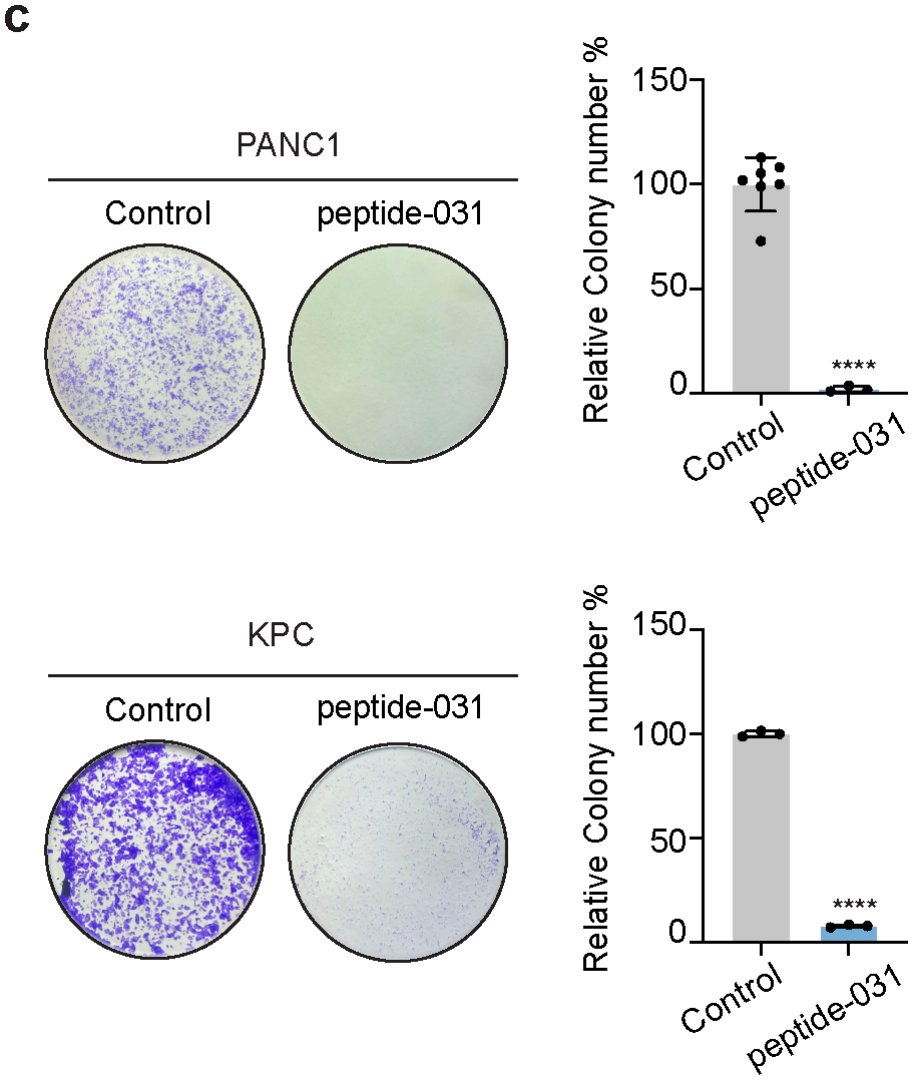


We sincerely apologize for these errors. The correction is approved by all authors of the original article. These corrections do not change the results and conclusions presented in the published article.

