# Supplementary material for: Inhibition of DEF‐p65 Interactions as a Potential Avenue to Suppress Tumor Growth in Pancreatic Cancer
Source: Adv Sci (Weinh). 2024 May 17;11(28):2401845. doi: 10.1002/advs.202401845 (PMC11267266; doi:10.1002/advs.202401845)
Supplement: Supplementary file 1 — Supporting Information [file ADVS-11-2401845-s001.docx]

**Supplementary Materials** **for**

**Inhibition of DEF-p65 Interactions as a Potential Avenue to Suppress Tumor Growth in Pancreatic Cancer**

**Sicong Huang**†, **Jiaqi Yang**†, **Teng Xie**†, Yangwei Jiang, Yifan Hong, Xinyuan Liu, Xuyan He, Damiano Buratto, Dong Zhang, Ruhong Zhou^*^, Tingbo Liang^*^, Xueli Bai^*^

**The file includes:**

Figure S1 to S6

Table S1 to S5


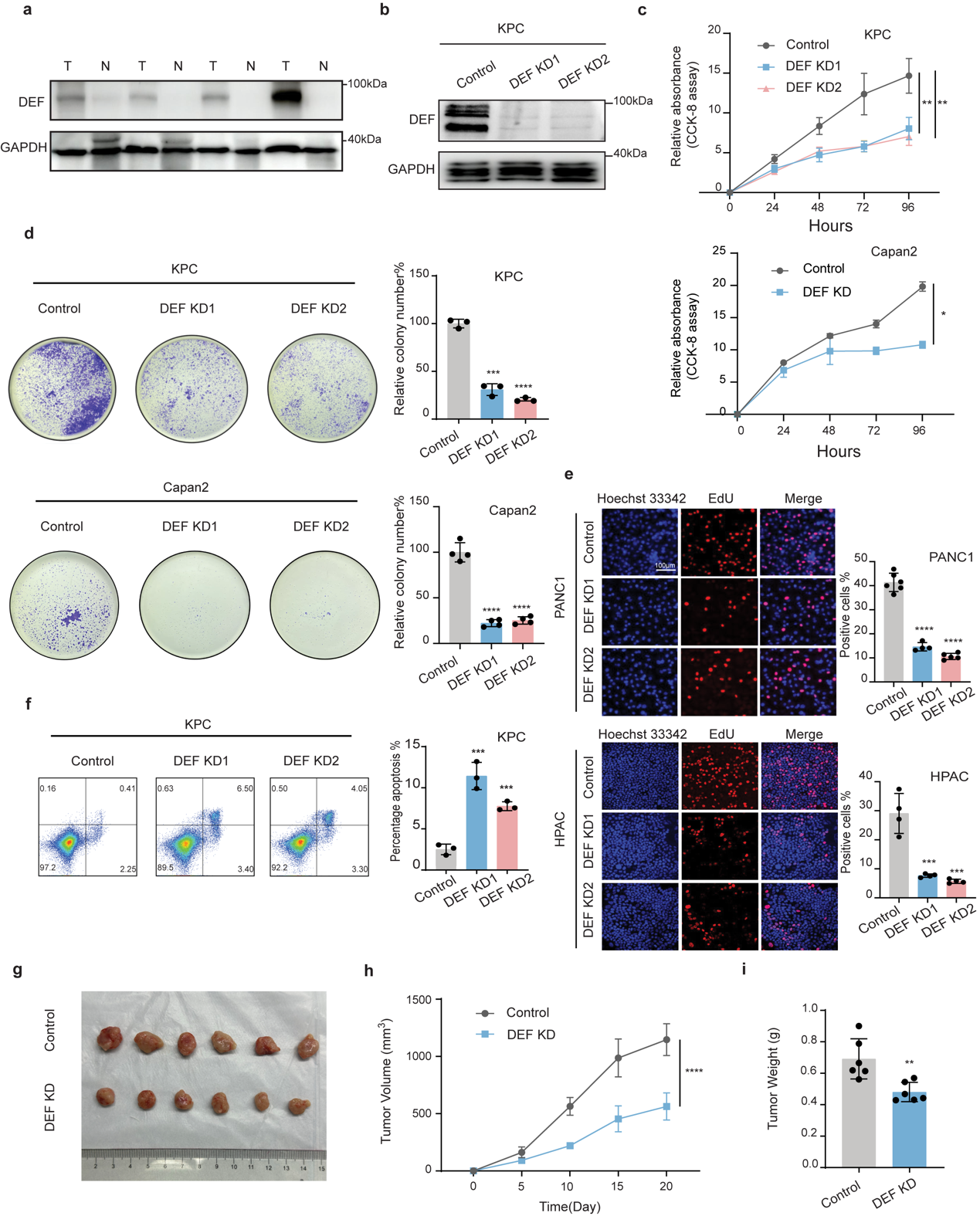


**Figure S1.** **DEF knockdown reduces the proliferation and induces apoptosis of PDAC cells *in vitro* and *in vivo*.** **a.** DEF expression was measured in paired tumor and normal pancreatic tissue by western blot (n=4). **b.** Western blotting analysis of control and DEF knockdown KPC cells to confirm knockdown. **c.** Cell growth of control and DEF knockdown in KPC and Capan2 cells was assessed by Cell Counting Kit-8 assay. **d.** Representative images and quantification of colony formation assays of DEF depleted pancreatic cancer cells. **e.** Representative images and histogram analysis of EdU assays after DEF knockdown in PANC1 and HPAC cells. Scale bars: 100 μm. **f.** Flow cytometric analysis of apoptosis in control and DEF knockdown cells. **g.** Gross appearance of xenograft tumors in immunodeficient nude mice after subcutaneous injections of control (n=6) and DEF knockdown KPC cells (n=6). **h-i.** Representative tumor volumes after injection (**h**) and mean tumor weights (**i**) on day 20. All the data were calculated by unpaired two-tailed Student’s *t-*tests. **p*<0.05, ***p*<0.01,****p*<0.001, *****p*<0.0001. Representative data from triplicate experiments are shown, and error bars represent SEM.


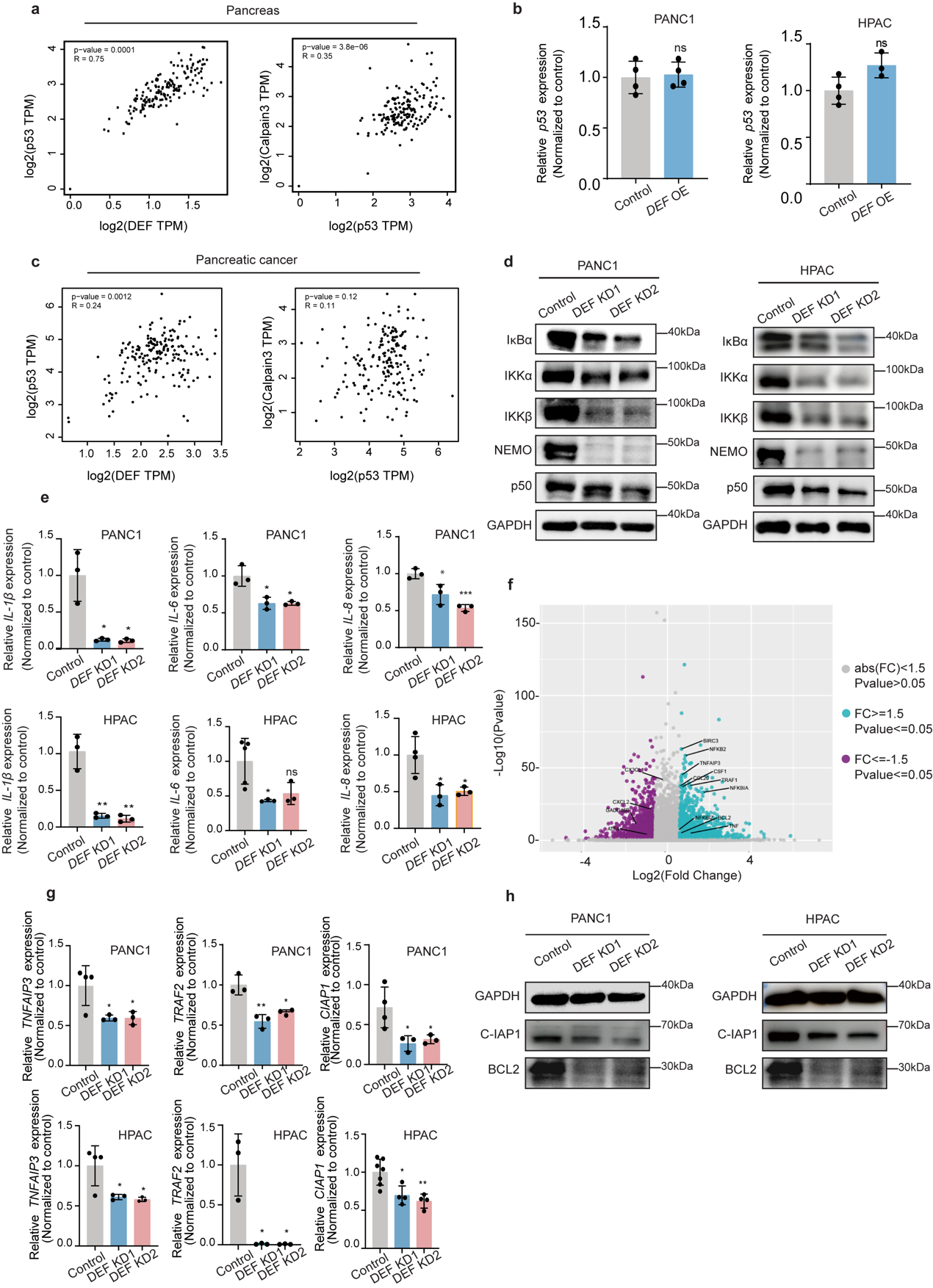


**Figure S2. DEF knockdown doesn’t affect the expression of p53 in pancreatic cancer cells, but by activating NF-κB pathway.** **a.** Correction of mRNA expression levels with *p53* and *DEF*, *Calpain3* in normal pancreas samples. **b.** RT-qPCR analysis of *p53* in control and *DEF* overexpression cell lines are shown. **c.** The plots show the correlation between *p53* and *DEF*, *Calpain3* expression in pancreatic cancer. RNA-seq data of pancreatic cancer patients and normal pancreas patients were analyzed from TCGA databases. Pearson correlation coefficient and one-tailed probability *p*-value are shown. **d.** Western blot analysis of NF-κB family members with control and DEF knockdown in PANC1 and HPAC cells is shown. **e.** The mRNA expression level of *IL-1β* , *IL-6*, and *IL-8* in control and *DEF* knockdown PANC1 (top) and HPAC cells (bottom) is shown. **f.** Volcano plot of genes upregulated (cyan) or downregulated (purple) in control (n=3) relative to *DEF* knockdown (n=3) PANC1 cells. **g.** RT-qPCR examination of *TNFAIP3*, *TRAF2*, and *C-IAP1* expression in pancreatic cancer cell PANC1 and HPAC with DEF knockdown. **h.** Western blotting analysis of C-IAP1 and BCL2 expression in DEF KD pancreatic cancer cells. All the data were calculated by unpaired two-tailed Student’s *t-*tests. ns, not significant, **p*<0.05,***p*<0.01,****p*<0.001, *****p*<0.0001. RT-qPCR, real-time quantitative polymerase chain reaction. Representative data from triplicate experiments are shown, and error bars represent SEM.


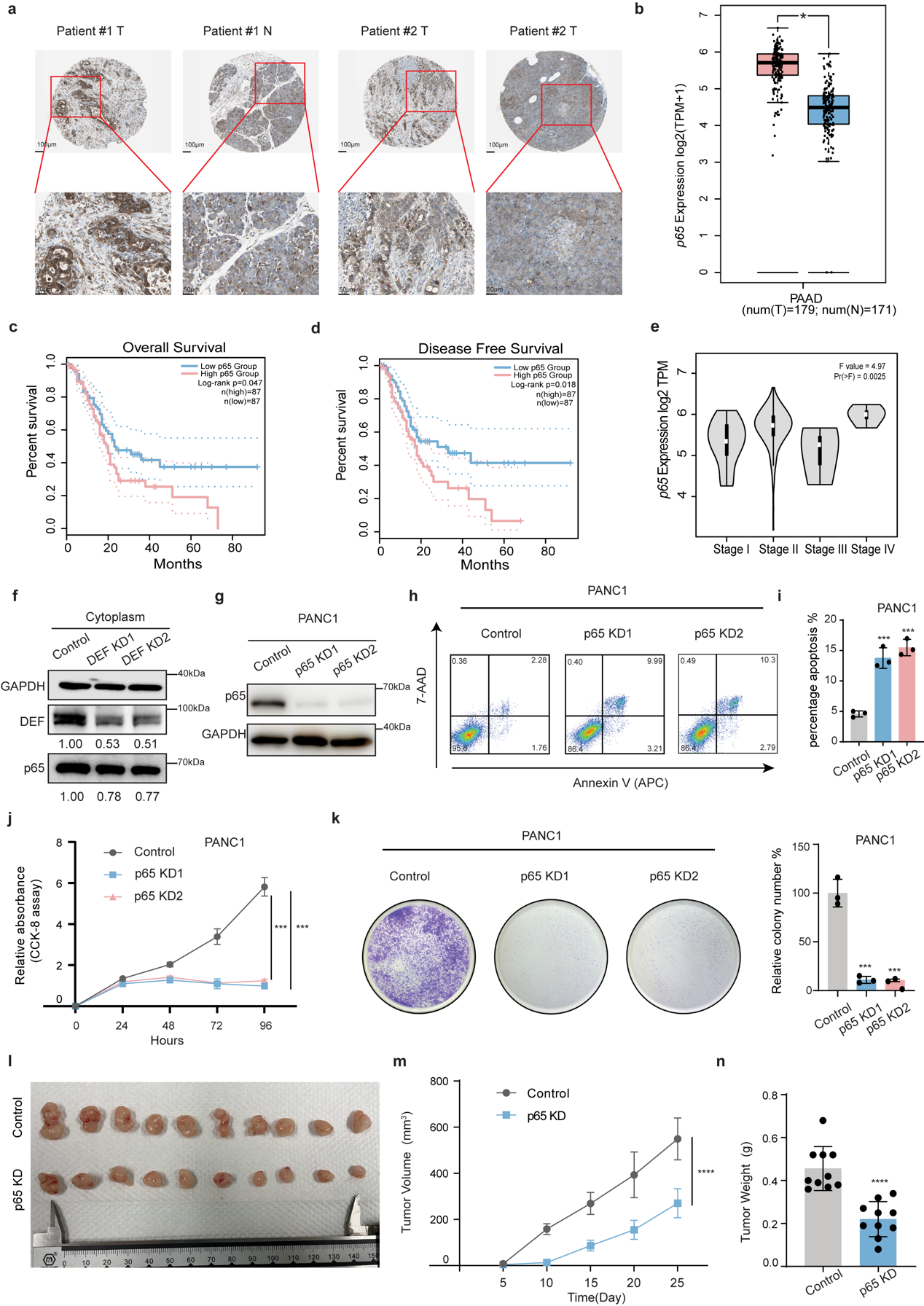


**Figure S3.** **p65 is involved in pancreatic cancer growth *in vitro* and i*n vivo*. a.** Representative images of IHC staining of p65 in a tissue microarray. Scale bar: 100 μm. **b.** The relative *p65* expression in pancreatic cancer and normal pancreatic tissues from the TCGA database (n=350). **c-d.** Overall survival (OS) and Disease free survival (DFS) of patients with pancreatic cancer with high or low concentrations of DEF (n=174). **e.** Differential expression of p65 between pancreatic tumor tissues with different clinical stages using TCGA dataset. **f.** Cytoplasmic and nuclear DEF and p65 protein levels after DEF knockdown in PANC1 cells. **g.** Western blotting analysis of p65 in p65 knockdown cells. **h-i.** Flow cytometric analysis was used to evaluate the incidence of apoptosis after p65 knockdown in PANC1 cells. **j.** Cell Counting Kit-8 assay comparing control and p65 knockdown cells are shown. **k**. Colony formation and statistical analysis of PANC1 cells. **l-n.** The visual map of the xenograft tumors after subcutaneous injections into immunodeficient mice with control and p65-depleted pancreatic cancer cells. Representative images of tumors (n=10) **(l)**, tumor volumes **(m)**, and tumor weights **(n)** were measured in the indicated groups. Tumors were measured at specified time points and dissected at the endpoints. Results are presented as mean ± SD from one representative experiment. **p*<0.05, ***p*<0.01,****p*<0.001, *****p*<0.0001 according to a two-tailed test*.* All data are representative of three independently performed experiments.


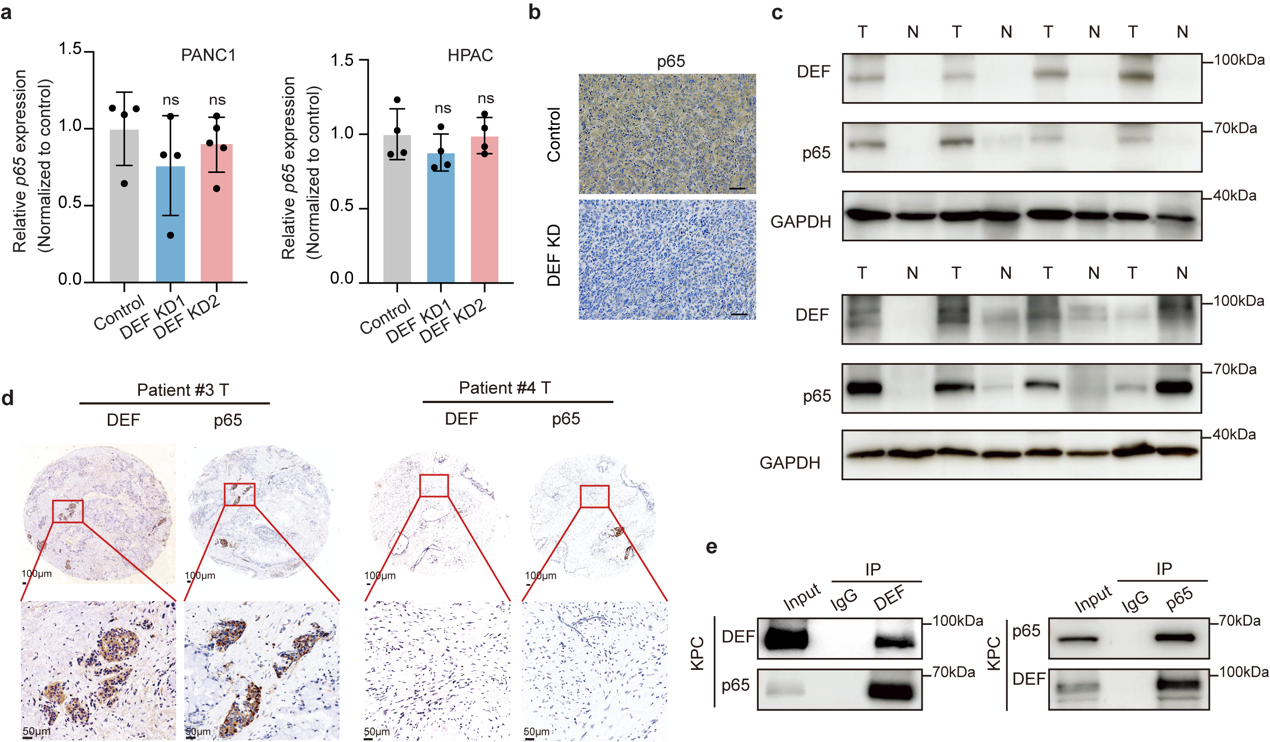


**Figure S4. DEF positively interacts with p65 in pancreatic cancer. a.** The mRNA expression level of *p65* in control and *DEF* knockdown PANC1 and HPAC cells is shown. **b.** Representative images of the results of p65 in DEF knockdown tumors within immunodeficient nude mice. Scale bars: 100 μm. **c.** Western blotting analysis of DEF and p65 in clinical pancreatic cancer tissue samples from patients (n=8) (T= pancreatic tumor tissue, N= normal pancreatic tissue). **d.** Representative images of IHC staining of DEF and p65 in a tissue microarray. Scale bars: 100 μm. **e.** Co-ip and western blot analysis of the endogenous DEF/p65 proteins interaction in the KPC. All the data were calculated by unpaired two-tailed Student’s *t-*tests. ns, not significant, **p*<0.05,***p*<0.01,****p*<0.001, *****p*<0.0001. RT-qPCR, real-time quantitative polymerase chain reaction. Representative data from triplicate experiments are shown, and error bars represent SEM.


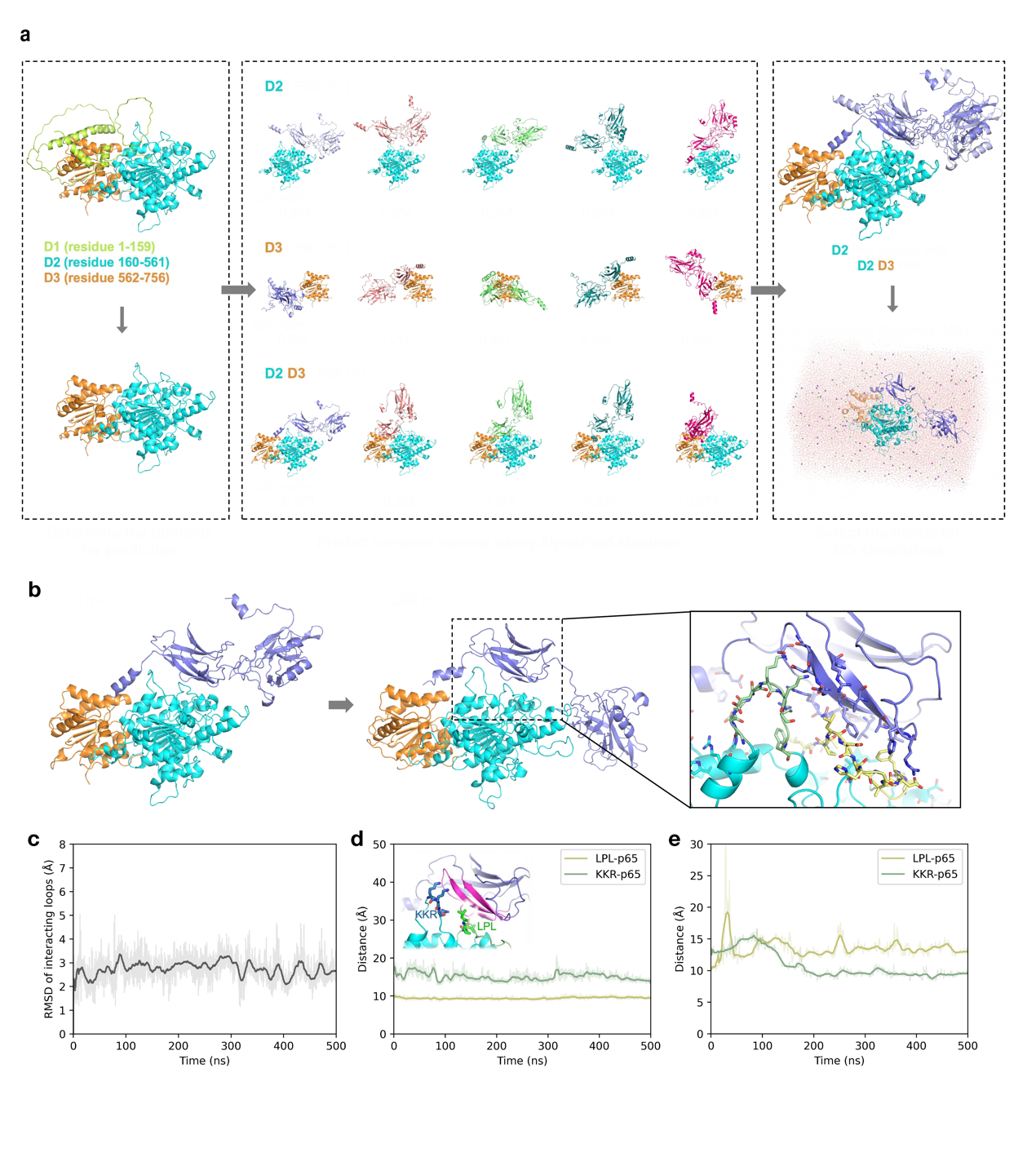


**Figure S5.** ***In silico* investigation on DEF/p65 interaction.** **a.** Prediction workflow for DEF/p65 complex. D2 (cyan), D3 (orange) and D2-D3 (cyan and orange) of DEF were selected to predict five models of complexes with P1 of p65 (different colors in the five models). The models were also ranked from left to right using the Alphafold-mulitmer model confidence (iptm+ptm). The top-ranked D2-D3/p65(P1) model was selected for MD simulations. **b.** Snapshot of the D2-D3/p65(P1) model during the MD simulation. The interface is shown in the enlarged view and two interacting loops of DEF are shown by sticks. **c.** RMSD of the two interacting loops of DEF after aligning p65 in the MD trajectory. **d.** Distance between the key interacting motif, LPL (green) or KKR (blue), the beta sheet (magenta) of p65 in D2-D3/p65 (P1) complex. **e.** Distance between LPL or KKR and p65 in peptide-031/p65 (P1) complex. The centers of mass of these motifs were used for the distance measurement.


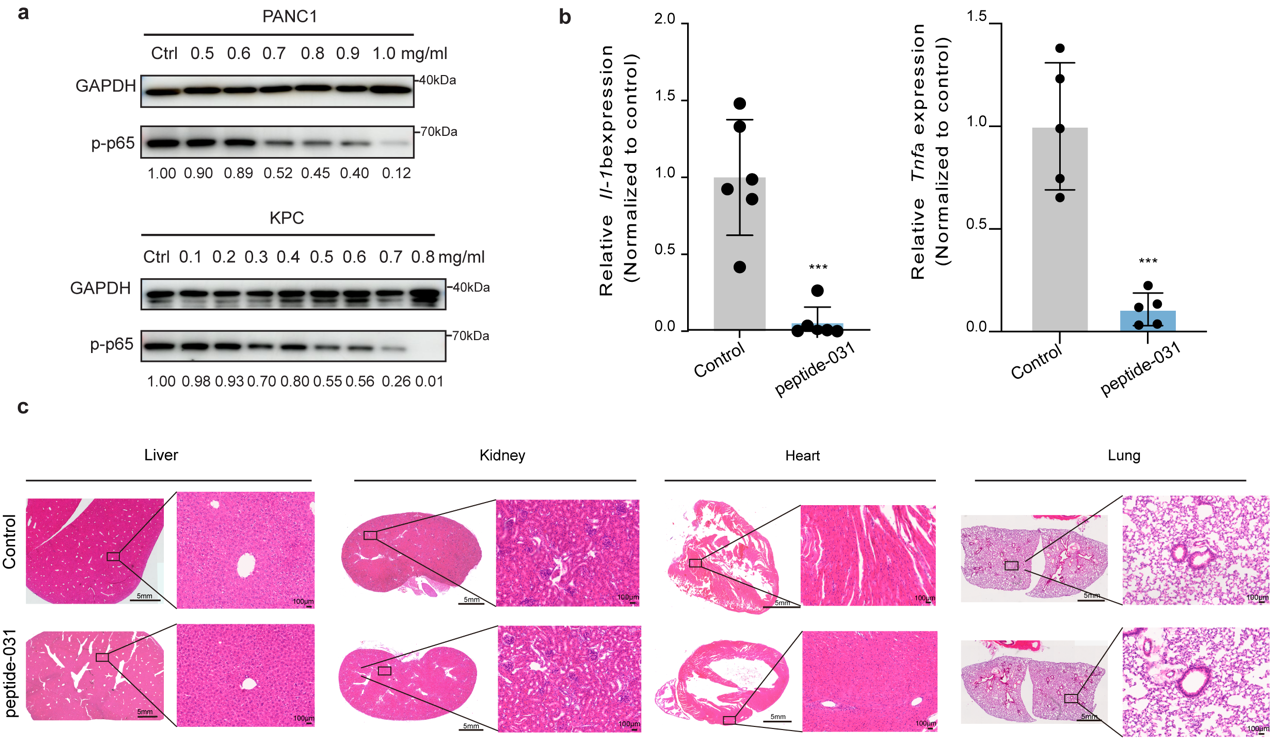


**Figure S6.** **Peptide-031 demonstrates a suppressive effect on the expression of phosphorylated p65 protein, as well as on the mRNA levels of IL-1β and TNFα in pancreatic cancer cells, and** **the peptide-031 exhibits a favorable safety profile.**

**a.** Western blot analysis of phosphorylated p65 of PANC1 and KPC cells treated with the indicated peptides at various concentrations for 24 h. **b.** Real-time quantitative polymerase chain reaction analysis of the *IL-1β* and *TNFα* in control and peptide-031 treatment KPC cells. **c.** Representative images show the effects of peptide-031 on the liver, kidney, heart, and lung tissues in mice, as revealed by H&E staining. Scale bars: 5 mm and 100 μm. Results are presented as mean ± SD. **p*<0.05, ***p*<0.01,****p*<0.001 according to a two-tailed *t*-test*.* All data are representative of three independently performed experiments.

**Table S1.** **Clinicopathological relevance of DEF in PDAC patients.**

| **Clinicopathological relevance of DEF in PDAC patients** | | | | |
| --- | --- | --- | --- | --- |
| **Variable** | **DEF** | | | |
|  | **Low (H-score ≤80）** | **High (H-score ＞80）** | **Chi-squre** | **P value** |
| **Gender** |  | |  | |
| Male, n(%) | 37(36.1) | 53(53.9) | \| 0.088 \| \| --- \| \|  \| | \| 0.767 \| \| --- \| \|  \| |
| Female, n(%) | 24(24.9) | 38(37.1) |  |  |
| **Age,years** |  | |  | |
| ≤60, n(%) | 16(16.6) | 25(24.4) | \| 0.044 \| \| --- \| \|  \| | \| 0.834 \| \| --- \| \|  \| |
| >60, n(%) | 45(44.4) | 65(65.6) |  |  |
| **TNM stage** |  | |  | |
| I, n(%) | 24(20) | 29(33) | \| 11.929 \| \| --- \| \|  \| \|  \| \|  \| | \| 0.008 \| \| --- \| \|  \| \|  \| \|  \| |
| II, n(%) | 26(23.8) | 37(39.2) |  |  |
| III, n(%) | 10(9.8) | 16(16.2) |  |  |
| IV, n(%) | 0(6.4) | 7(10.6) |  |  |
| **Vascular invasion** |  | |  | |
| Yes, n(%) | 28(30.2) | 47(44.8) | \| 0.541 \| \| --- \| \|  \| | \| 0.462 \| \| --- \| \|  \| |
| No, n(%) | 32(29.8) | 42(44.2) |  |  |
| **Lymphatic metastasis** |  | |  | |
| Yes, n(%) | 25(31) | 52(46) | \| 4.032 \| \| --- \| \|  \| | \| 0.045 \| \| --- \| \|  \| |
| No, n(%) | 35(29) | 37(43) |  |  |
| **CA19-9,U/mL** |  | |  | |
| ≤37, n(%) | 11(11.4) | 18(17.6) | \| 0.035 \| \| --- \| \|  \| | \| 0.851 \| \| --- \| \|  \| |
| >37, n(%) | 47(46.6) | 71(71.4) |  |  |
| **Distant metastasis** |  |  |  |  |
| Yes, n(%) | 1(3.6) | 8(5.4) | \| 3.386 \| \| --- \| \|  \| | \| 0.066 \| \| --- \| \|  \| |
| No, n(%) | 59(56.4) | 81(83.6) |  |  |

**Table S2. FEP results of relative binding free energy difference (ΔΔG) of mutations in DEF residues at the interface.**

|  | K465A | K466A | R467A | **KKR-AAA** |
| --- | --- | --- | --- | --- |
| **ΔΔG（kcal/mol）** | 3.44±0.18 | 0.52±0.12 | 0.96±0.17 | 3.74±0.31 |

|  | L504A | L506A | **LPL-AAA** |
| --- | --- | --- | --- |
| **ΔΔG**  **（kcal/mol）** | 3.82±0.09 | -1.23±0.11 | 1.53±0.33 |

**Table S3. List of primers used for RT-qPCR.**

| **Primer Name** | **Primer sequence** |
| --- | --- |
| DEF-F | GCCTCTCAAGATCCATTTCTTCA |
| DEF-R | AAAGCTGGCCCAGAATAGGC |
| GAPDH-F | GGAGCGAGATCCCTCCAAAAT |
| GAPDH-R | GGCTGTTGTCATACTTCTCATGG |
| p53-F | TGACACGCTTCCCTGGATTG |
| p53-R | GCTCGACGCTAGGATCTGAC |
| TNFα-F | GCAACAAGACCACCACTTCG |
| TNFα-R | CTGGAGGCCCCAGTTTGAAT |
| TRAF2-F | GCGCTGCGACCGTTG |
| TRAF2-R | ACTTGGCTTCCAGCTTGGTC |
| TNFAIP3-F | TCAACTGGTGTCGAGAAGTCC |
| TNFAIP3-R | CAAGTCTGTGTCCTGAACGC |
| IL-1β-F | TTGTTGCTCCATATCCTGTCCCT |
| IL-1β-R | CCCTTCATCTTTGAAGAAGAACC |
| IL-6-F | GGCACTGGCAGAAAACAACC |
| IL-6-R | GCAAGTCTCCTCATTGAATCC |
| IL-8-F | ACACTGCGCCAACACAGAAATTA |
| IL-8-R | TTTGCTTGAAGTTTCACTGGCATC |
| c-IAP2-F | TCCTGGATAGTCTACTAACTGCC |
| c-IAP2-R | GCTTCTTGCAGAGAGTTTCTGAA |
| Def-F | GAAATCGCAGCTTAGCCCAG |
| Def-R | TGGTACCCTGAAACGTGCTC |
| Gapdh-F | ATCATCCCTGCATCCACT |
| Gapdh-R | ATCCACGACGGACACATT |
| Il-1β-F | GGTACATCAGCACCTCACAA |
| Il-1β-R | TTAGAAACAGTCCAGCCCATAC |
| Tnfα-F | TTGTCTACTCCCAGGTTCTCT |

**Table S4. Table of shRNA sequences used.**

| **shRNA** | **Target sequence (5’-3’)** |
| --- | --- |
| shRNA Control | CCTAAGGTTAAGTCGCCCTCG |
| shRNA DEF #1 | GCACTGTTCTCTACTCCAAAT |
| shRNA DEF #2 | CTTCGTGCGTCTTCGAAATTA |
| shRNA p65 #1 | CGGATTGAGGAGAAACGTAAA |
| shRNA p65 #2 | ATGGATTCATTACAGCTTAAT |
| shRNA Def #1 | CTTTGTGCGTCTCCGAAATTA |
| shRNA Def #2 | ACCTAGAAGAGTACGTTTATT |

**Table S5. Table of key resources.**

| **Antibodies** | **Source** | **Identifier** |
| --- | --- | --- |
| GAPDH | CST | 5174 |
| p53 | CST | 2527 |
| Histone H3 | CST | 4499 |
| p50 | Abcam | ab32360 |
| p-p65 | CST | 3033 |
| DEF | ThermoFisher | A305-122 |
| DEF | HuaBio | NA |
| IκBα | CST | 4814 |
| IKKα | Abcam | 32041 |
| IKKβ | CST | 4814 |
| NEMO | Abcam | ab178872 |
| Flag | CST | 14793 |
| His | Sigma | SAB1305538 |
| BCL2 | CST | 15071 |
| C-IAP2 | CST | 7065 |
| p65 | Abclonal | A10609 |
| p65 | CST | 8242 |
| p65 | Abcam | ab32536 |
| c-caspases 3 | CST | 9664 |
| Ubiqutin | CST | 3933 |
| IL-6 | Servicebio | GB11117 |
| IL-1β | Servicebio | GB11113 |

| Ki-67 | eBioscience | 14-5698-82 |
| --- | --- | --- |
